# Birth in Brazil: national survey into labour and birth

**DOI:** 10.1186/1742-4755-9-15

**Published:** 2012-08-22

**Authors:** Maria do Carmo Leal, Antônio Augusto Moura da Silva, Marcos Augusto Bastos Dias, Silvana Granado Nogueira da Gama, Daphne Rattner, Maria Elizabeth Moreira, Mariza Miranda Theme Filha, RosaMariaSoaresMadeira Domingues, Ana Paula Esteves Pereira, Jacqueline Alves Torres, Sonia Duarte Azevedo Bittencourt, Eleonora D’orsi, Antonio JLA Cunha, Alvaro Jorge Madeiro Leite, Rejane Silva Cavalcante, Sonia Lansky, Carmem Simone Grilo Diniz, Célia Landmann Szwarcwald

**Affiliations:** 1 Escola Nacional de Saúde Pública Sergio Arouca, Fundação Oswaldo Cruz, Rio de Janeiro, Brasil; 2 Universidade Federal do Maranhão, Maranhão, Brazil; 3 Instituto Fernandes Figueira, Fundação Oswaldo Cruz, Rio de Janeiro, Brazil; 4 Universidade Nacional de Brasília, Brasília, Brasil; 5 Agência Nacional de Saúde Suplementar, Rio de Janeiro, Brazil; 6 Universidade Federal de Santa Catarina, Santa Catarina, Brazil; 7 Universidade Federal do Rio de Janeiro, Rio de Janeiro, Brazil; 8 Universidade Federal do Ceará, Ceará, Brazil; 9 Universidade Federal do Pará, Pará, Brazil; 10 Secretaria Municipal de Saúde de Belo Horizonte, Belo Horizonte, Brazil; 11 Universidade de São Paulo, São Paulo, Brazil; 12 Instituto de Comunicação e Informação Científica e Tecnológica em Saúde, Fundação Oswaldo Cruz, Rio de Janeiro, Brazil; 13 Escola Nacional de Saúde Pública Sergio Arouca, Rua Leopoldo Bulhões, 1480, sala 809 - Manguinhos, Rio de Janeiro, RJ, Brazil, CEP: 21041-210

**Keywords:** Caesarean section, Elective Caesarean section, Robson’s groups, Near miss maternal morbidity, Near miss neonatal morbidity, Prematurity, Low birth weight, Brazil

## Abstract

**Background:**

Caesarean section rates in Brazil have been steadily increasing. In 2009, for the first time, the number of children born by this type of procedure was greater than the number of vaginal births. Caesarean section is associated with a series of adverse effects on the women and newborn, and recent evidence suggests that the increasing rates of prematurity and low birth weight in Brazil are associated to the increasing rates of Caesarean section and labour induction.

**Methods:**

Nationwide hospital-based cohort study of postnatal women and their offspring with follow-up at 45 to 60 days after birth. The sample was stratified by geographic macro-region, type of the municipality and by type of hospital governance. The number of postnatal women sampled was 23,940, distributed in 191 municipalities throughout Brazil. Two electronic questionnaires were applied to the postnatal women, one baseline face-to-face and one follow-up telephone interview. Two other questionnaires were filled with information on patients’ medical records and to assess hospital facilities. The primary outcome was the percentage of Caesarean sections (total, elective and according to Robson’s groups). Secondary outcomes were: post-partum pain; breastfeeding initiation; severe/near miss maternal morbidity*;* reasons for maternal mortality; prematurity; low birth weight; use of oxygen use after birth and mechanical ventilation; admission to neonatal ICU; stillbirths; neonatal mortality; readmission in hospital; use of surfactant; asphyxia; severe/near miss neonatal morbidity. The association between variables were investigated using bivariate, stratified and multivariate model analyses. Statistical tests were applied according to data distribution and homogeneity of variances of groups to be compared. All analyses were taken into consideration for the complex sample design.

**Discussion:**

This study, for the first time, depicts a national panorama of labour and birth outcomes in Brazil. Regardless of the socioeconomic level, demand for Caesarean section appears to be based on the belief that the quality of obstetric care is closely associated to the technology used in labour and birth. Within this context, it was justified to conduct a nationwide study to understand the reasons that lead pregnant women to submit to Caesarean sections and to verify any association between this type of birth and it’s consequences on postnatal health.

## Background

Caesarean section rates in Brazil have been steadily increasing. Estimates from the 1970s indicate that the rate of caesarean section births was around 15%, rose to 38% in 2001 and to 48.8% in 2008. In 2008, 35% of births within the Unified Health System and 80% of births in the private sector were Caesarean [1]. In 2009, the Caesarean rate was 50.1%, and for the first time the number of Caesarean section was greater than the number of vaginal births [2].

Risks to women, associated to Caesarean births, are becoming increasingly evident. A survey conducted in eight countries in Latin America, showed that increased Caesarean rates are associated with an increase in maternal mortality, higher use of antibiotics in the post-partum period and severe maternal morbidity, even after adjusting for confounding factors [3]. Another prospective study also showed higher frequencies of maternal mortality, hospitalization in intensive care unit (ICU), blood transfusion, hysterectomy, antibiotic therapy and longer hospital stay for women who underwent elective Caesarean section [4].

Caesarean section is a well-established risk factor to the subsequent development of an abnormal placentation [5], increased prevalence of postpartum fever [6], as well as higher risk of uterine rupture, post-partum hemorrhaging, manual removal of the placenta, infection and admittance to ICU [7].

Caesarean section is also associated with a series of adverse effects on the newborn, including: higher rates of neonatal mortality [4], intermediate (32–33 weeks) and late (34–36 wks) pre-term births, admittance to neonatal intensive care unit (NICU)[8] and use of mechanical ventilation in at term newborn from low-risk pregnancies [9,10]. In a retrospective cohort with 2693 late preterm births found that over 50% of them were born by Caesarean section, with no scientific evidence for recommending them and in which the procedure was closely associated to admissions of the newborn in the NICU [10].

Although Caesarean sections can reduce mortality in extremely preterm newborns (from 22 to 25 weeks of pregnancy) [11], it appears to be associated with higher neonatal mortality amongst those born with 32 to 36 weeks of pregnancy [12].

Tomashek et al. and Swamy et al. found that late pre-term newborns (34 to 36 weeks of pregnancy) had higher mortality rates during childhood, with adverse outcomes not limited to immediate complications after birth [13,14].

The evaluation of the relationship between Caesarean section and preterm birth in Brazil is limited by the quality of data available in the national Live Birth Information System - SINASC, especially in the North and Northeast regions and in smaller cities [15]. Official data shows a very low prematurity rate, at 6.6%, more than 50% below rates described in epidemiological studies published in Brazil, which suggests that the prematurity rates based on the SINASC are underestimated in many regions of the country [16-18].

Nevertheless, recent evidence suggests that the increasing rates of prematurity and low birth weight in Brazil are associated to the increasing rates of Caesarean sections and induced vaginal births. Data from a cohort study in Pelotas, a mid-sized town in southern Brazil, showed that the prematurity rate in that municipality increased from 6.3% in 1982 to 11.4% in 1993 and to 14.7% in 2004. Concomitantly, there has been an increase in Caesarean births (28%, 31% and 45%) and the use of instrumental techniques for vaginal births, within a scenario of excessive medical interventions [19]. Cohort data from Ribeirão Preto, a city within the state of São Paulo, also showed that the prematurity rate increased from 7.6% in 1978/79 to 13.6% in 1994, and has been associated to an increase in the rate of Caesarean sections [16,20,21].

Studies conducted in Brazil have been unable to confirm the hypothesis of increased Caesarean sections due to women’s “demand” [22]. One investigation conducted in the postnatal ward in two hospitals from the private sector in the metropolitan region of Rio de Janeiro state found that over 70% of multiparous pregnant women and 80% of nulliparous pregnant women desired to have a vaginal birth at the beginning of their pregnancies. Nonetheless, by the end of the pregnancy, at the time of birth, only 30% of them maintained this wish, and, at the end of pregnancy, only 10% of these women had vaginal births. The reasons given by the women for having had Caesarean sections did not coincide with the indication written on the medical records, nor with results observed throughout the pregnancy [22]. Similar results were obtained in São Luis, capital of the state of Maranhão in northeastern Brazil [23]. The change in the type of birth, in relation to the initial desire, seems to have been influenced by the interventionist conduct of the clinician [24]. If a Caesarean section was decided upon after hospitalization, this has already been described in literature as a clinical entity - “Intrapartum elective cesarean delivery” - where the profile of the attending clinician is the main determinant for the decision to perform a Caesarean section before any concrete obstetrical indication [25].

One observes, in relation to women in Brazil, that preference for Caesarean sections is associated with higher socioeconomic level, white ethnicity, higher education and higher adequacy of antenatal care [26-28]. Regardless of socioeconomic level, demand for Caesareans appears to be based on the belief that quality of obstetric care is closely associated to the technology used in the surgical birth [29].

Within this context, it would be justified to conduct a nationwide study to understand the reasons that lead pregnant women to submit to Caesarean sections, to verify any association between this type of birth and postnatal health consequences to mother and newborn, including premature birth and low birth weight, focusing particularly on late prematurity (34 to 36 weeks of pregnancy).

The aims of the *Birth in Brazil: national survey into labour and birth* study are

1- To describe the incidence of excessive caesarean section (according to Robson’s groups) and examine the consequences on women’s and newborn’s health;

2- To investigate the relationship between excessive caesarean section and late preterm birth and low birth weight;

3- To investigate the relationship between excessive caesarean section and the use of technological procedures after birth.

## Methods

### Study design and population

A nation wide hospital-based cohort study, with follow-up of post-partum women and newborn health at 45 to 60 days after birth. The study received funding from the National Research Council - Ministry of Science and Technology of Brazil and from the Oswaldo Cruz Foundation (FIOCRUZ) of the Ministry of Health.

### Time period of the study

Data collection started in February 2011 and finished in July 2012. Data is being prepared for analyses.

### Inclusion criteria

Hospitals that recorded 500 births or more were eligible (SINASC, 2007) to enter in the study sample. Postnatal women who gave birth to a live newborn, regardless of weight or gestational age, or to a stillbirth with birth weight ≥500 g and/or gestational age ≥22 weeks of pregnancy in one of the eligible hospitals were invited to participate in this study.

### Exclusion criteria

The study excluded women who delivered at home, or with severe mental health disorder, who were homeless or foreigners who did not understand Portuguese language; deaf/mutes; and women sectioned by court order.

### Recruitment strategies

From each hospital included in the study recruited 90 postnatal women and their offspring. The time spent by the research team in each hospital varied according to the number of births per day. In hospitals with less than 12 births per day, all postnatal women were included until a total of 90 mother and baby pairs were reached. In those units with a larger number of births, we ensured that the data collection period accounted for every day of the week, including weekends and holidays. In these hospitals, postnatal women were randomly selected from a list of daily admittances, which included all daytime and nighttime births.

Women and newborn who remained in hospital were tracked by the study for as long as 28 days (for newborn) and 42 days (for women), including those transferred to other hospitals.

A pilot study was conducted in two municipalities in the Northeast and Southeast regions, to test and adjust the questionnaire and for data collection logistics.

### Sample design

Caesarean section rates vary according to the geographic location and characteristics of the clientele. Thus, the sample was stratified by geographic macro-region (North, Northeast, Southeast, South and Mid-West), type of the municipality (capital and no capital cities), and by type of hospital governance (public, private or mixed financing). Hospitals that were listed in the National Health Establishment Information System as private but that had bed vacancies contracted by the public sector were classified as mixed financing.

Six strata were produced for each of the five macro-regions: capital and no capital cities, Private/Mixed/Public. In the end, our sample was made up of 30 strata.

A probabilistic sample in two stages was selected for each stratum. Firstly, hospitals were selected and then postnatal women and their offspring.

A total of 1,403 of the 3,961 hospitals registered in the country in 2007 were eligible for the study (500 or more births) and 78.6% of births that year took place in them.

The sample size in each stratum was calculated based on the Caesarean section rate in Brazil in 2007 of 46.6%, with 5% significance to detect differences of 14% (difference between mixed and private hospitals) and with testing power of 95%. The minimum sample per stratum was 341 postnatal women. Since the sample was by conglomerates, a design effect of 1.3 was used, reaching a minimum sample size of 444 ≈ 450 postnatal women per stratum. Moreover, the sample size has a power of 80% to detect adverse outcomes in the order of 3%, and differences of at least 1.5% among large geographic regions or type of hospital governance (public/private/mixed).

A total of 266 hospitals were sampled for the study, 19% of all those with 500 births or more in 2007. The number of hospitals sampled for each stratum complied to proportional allocation, with the minimum number of 5 hospitals in each stratum, varying from 5 to 39. In the Northern Region, the “no capital city-private” could not be formed, due to the absence of availability of this category of hospital.

**Figure 1:**
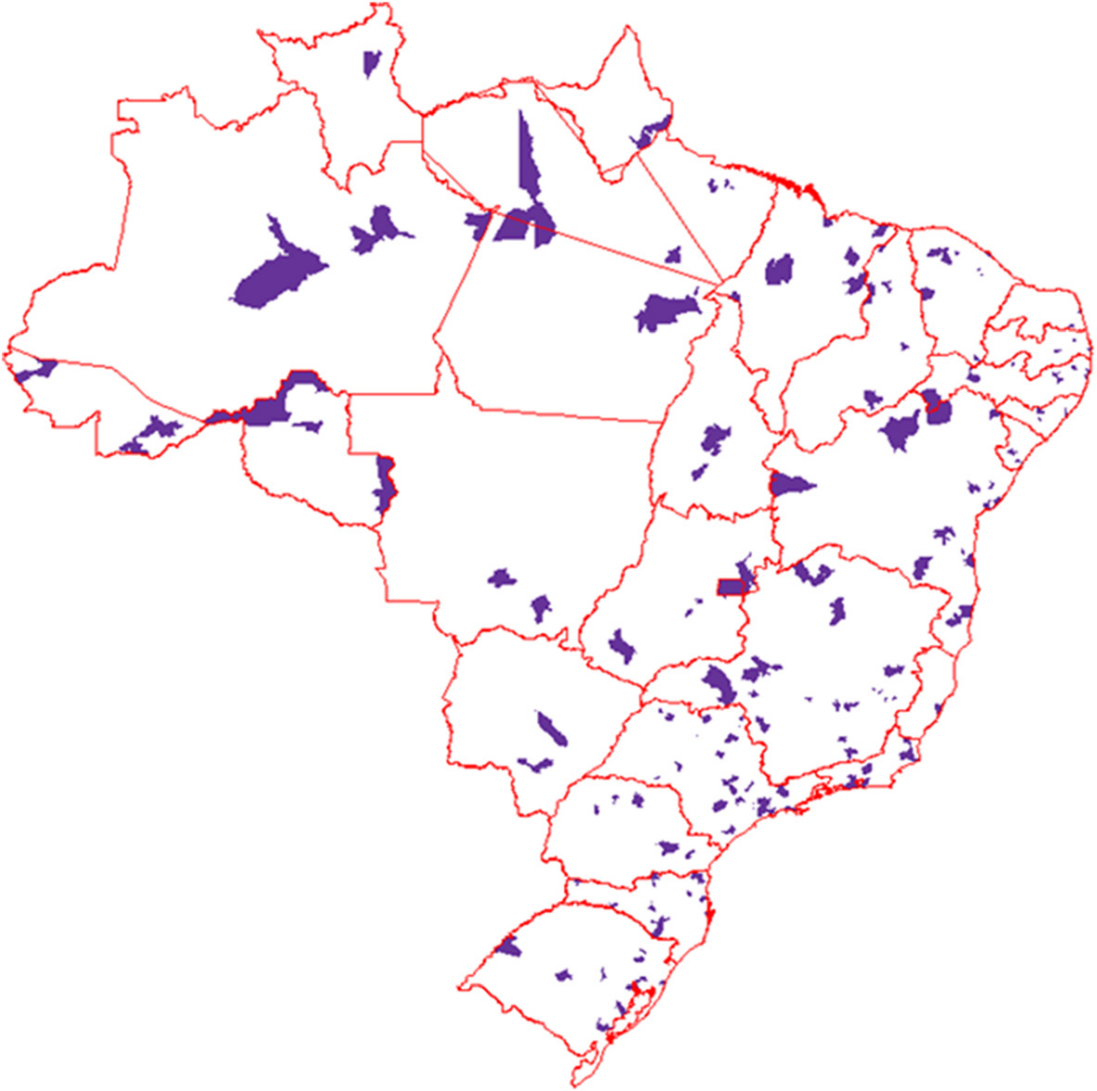
Distribution of municipalities throughout Brazil with at least one hospital in the study sample.

The number of postnatal women sampled was 23,940, distributed in 191 municipalities throughout Brazil, with 27 of these in state capitals and 164 in the rural areas and including every state in the country (Figure 1).

### Measurement instruments

Electronic forms (touch screen questionnaires) were developed and validated to collect data. The first questionnaire was completed with information about the woman at the health care unit within the first 24 hours after birth. The questionnaire was made up of variables with the mother’s identification details, educational and income levels, living conditions, prior birth history, maternal anthropometric data, information on current pregnancy, antenatal care, obstetric background, illnesses and use of medication during pregnancy, labour and assessment of care provided to her and to the newborn. An additional documentation file shows this questionnaire in detail [see Additional file 1]. At the interview, antenatal records of pregnant women and their antenatal ultrasound exams were photographed for later retrieval of specific data, with information retrieved in its own form.

The second questionnaire was completed with data available on the patients’ medical records, after discharge from hospital or on the 42nd day for the woman and the 28th day for the newborn that remained in hospital. Data was obtained on antenatal care, hospital admission, labour and birth information, medication and interventions performed, in addition to how the birth outcomes?: a) for the woman (type of birth, dilatation at time of admission to hospital, spontaneous or induced labour, use of pain relief and anaesthesia during labour, immediate complications from anaesthesia, partogram use, oxytocin use during labour, cardiotocography at admission and in labour, amniotomy, episiotomy, fundal pressure [Kristeller maneuver], presence of a family member [companionship during labour, birth and postnatal period] and maternal morbidity); b) for the newborn (Apgar score, weight at birth, gestational age, immediate post-birth care, use of oxygen and mechanical ventilation, clinical intercurrences, admission to NICU or intermediate care unit [IMCU], initiation of breastfeeding); c) conditions at hospital discharge or death (cause) of mother and offspring. An additional documentation file shows this questionnaire in detail [see Additional file 2].

Another interview was undertaken with all women between 45 and 60 days after birth, by telephone, to obtain information on: a) mother – re-hospitalization, puerperal complications, maternal discomfort, satisfaction with care received and reassessment of reason for opting for Caesarean section; b) newborn - breastfeeding, immunization, morbidity, re-hospitalization and death [see Additional file 3].

Furthermore, another questionnaire on a paper form was given by the supervisor to the management of the hospital in order to assess hospital facilities: no. of beds, professional staff (quantitative by specialty), hospital certification (teaching, reference for high risk, BFI - Baby Friendly Hospital Initiative - or other), availability of clinical pathology laboratory, blood transfusion unit, human milk bank, adult and neonatal IMCU/ICU, compliance to evidence-based protocol and appropriate use of labour and birth technology.

Field research supervisors reapplied the questionnaire to a random sample of 5% in the interviews with the women.

Telephone interviews were undertaken by a company with recognized competence in this type of data collection. Loss to follow-up was considered? when the woman stated that she had no telephone or when they could not be contacted after five attempts on alternate times and days.

Manuals were prepared with descriptions of procedures for selecting study participants, in large hospitals, and for data collection in order to ensure the quality of data and thereby minimize systematic or random errors.

### Administering the survey and data management

The research team was comprised of an executive coordination, including 10 researchers from different Brazilian teaching and research institutions. Each Brazilian macro region had a designated regional research coordinator and each Brazilian state a designated state research coordinator, who participated in organizing field work and in selecting the state’s team of research supervisors (50 in number) and interviewers (200 in number).

Training of the 27 state research teams was performed locally, in a standardized manner, over five consecutive days, including questionnaire instrument reading, practical use of the questionnaire in hospitals and sending data collected to the FIOCRUZ central server.

Each interviewer and supervisor received a login and password before beginning the study’s field work, which were stored together with the data from interviews/ medical records and enabled identification of the persons responsible for filling out the questionnaire.

The questionnaire presented a single identification with a code for the state, a code for the municipality, a code for the hospital, type of questionnaire and number with the order of the postnatal woman in the unit. Identification of the hospital allowed us to link it to the substrate to which it pertained: geographic region, unit location and type of hospital.

In order to avoid errors, the following procedures were adopted:

1- Answers that could not be entered because the data entry program blocked invalid values;

2- During the pilot-test phase, we sought to identify all possible answers that would cover the regional diversity of the country, since the study was nationwide in scope;

3- Any questions that were not applicable due to the answers given in the previous question did not appear on the screen to the interviewer, avoiding improper response to irrelevant questions and making the interview run more quickly;

4- The questions were stored at the end of each screen and if any were not correctly filled in or left blank, the program would not allow the interview to continue, flagging the questions that had problems;

5- Completed questionnaires remained in the program itself until exported. The data was exported initially in the netbook itself, in a working area on the desktop. Later these files were saved on a USB drive and handed to the research supervisor of each unit, responsible for sending the questionnaires to the central research site located at the FIOCRUZ server in Rio de Janeiro, where all the data was stored.

Online access to the database enabled real time monitoring of the field work by coordination.]

### Ethical aspects

The protocol was submitted to and approved by the Research Ethics Committee at the National School of Public Health - FIOCRUZ/Ministry of Health (opinion no. 92/10).

Before beginning the interview a Term of Free and Informed Consent was read. Consent was obtained on a digital medium, with the woman receiving a printed version, containing identification and contact details of the research coordinators. Whenever there was a refusal, the interviewee was invited to fill out a small form of refusals and losses, which inquired about age, educational level, ethnicity, type of birth and whether they had private health coverage or not.

### Outcome variables

I - For pregnant women: percentage of Caesarean sections (total; by group, according to Robson’s criteria [30]; and at the request of the woman); percentage of women with postpartum pain; percentage of newborn breastfed in the first hour of life; percentage of women with severe/near miss maternal morbidity*;* Reasons for maternal mortality. II – Perinatal and late neonatal outcomes: percentage of preterm (<37 weeks pregnancy) and late preterm births (34 to <37 weeks pregnancy); percentage of low birth weight (<2500 g); percentage of the use of oxygen after birth; percentage of the use of mechanical ventilation; percentage of admission to NICU; rate of intrapartum fetal mortality (stillbirths); rate of early (0–6 days) and late (7–27 days) neonatal mortality; percentage of readmission in hospital; percentage of surfactant use; percentage of moderate to severe asphyxia; percentage of severe/near miss neonatal morbidity.

Robson’s groups establish an expected proportion of Caesarean sections in ten groups defined according to the obstetric characteristics of the pregnant woman: prior birth, prior Caesarean section, single or multiple pregnancy, gestational age, type of fetal presentation, type of labour (spontaneous or induced), time of Caesarean performed (prior to or during labour). These categories are mutually exclusive, include all obstetric conditions, and are clinically relevant and retrospectively identifiable. They allow comparison between different services, estimate the excess of Caesarean sections in each category, providing inputs for change in care practices in different groups of women [31].

We defined elective Caesarean section as those with no spontaneous or induced labour and Caesarean sections for non-clinical indications as those decided at the beginning or at the end of pregnancy by exclusive choice of the woman or in conjunction with the attending clinician, when motivated by reasons not related to the obstetric or clinical condition.

We adopted the WHO criteria for identification of maternal near miss cases [32].

### Gestational age definition

Gestational age at birth (GA) was primarily calculated based on early ultrasound (US) performed between weeks 7 and 14 of pregnancy. Last menstrual period (LMP) and somatic scores (Capurro e Ballard) will also be used. Given the possibility of error of the GA calculated by these latter methods [33], when birthweight is incompatible with GA, in other words, when it is above the 99th percentile or below the 1^st^ percentile of the Canadian curve [34], the GA will be recoded as ignored. The same procedure were followed in cases of implausible GA (less than 20 or greater than 45 weeks). Cases of ignored GA were imputed in a regression model with the following predictive variables: birthweight, parity sex of newborn and mother’s education [35,36].

### Near miss neonatal morbidity indicator

The severe near miss neonatal morbidity indicator was developed based on comparison of newborns that died during the neonatal period and those that survived, using logistic regression. Odds ratios were estimated to identify situations closely associated to the risk of death. The intent was to identify situations predictive of neonatal death that can form part of a severe near miss neonatal morbidity indicator. This indicator was then used to assess whether Caesarean section is associated to an increase in severe near miss neonatal morbidity. The variables used as possible near miss predictors were: Apgar score less than 7 in the 5th minute of life, gestational age in weeks (≤ 33, 34 a 36 e ≥37), weight at birth in grams (< 1500, 1500 to 2499 and ≥ 2500), use of mechanical ventilation, use of oxygen after birth, occurrence of respiratory morbidity in the newborn, hypoglycemia and others [37].

### Intervening variables

The following were considered as intervening variables in the analysis of the outcomes: Socioeconomic class - A (highest), B, C, D and E (lower), according to the Brazil economic classification of the Brazilian Association of Research Entities – ABEP/2010) [38]; mother’s education; ethnicity (self-defined, according to categories used by IBGE)[39]; self-reported anthropometry (pre-pregnancy weight and height of woman); Maternal habits – alcohol consumption, smoking habits before and during pregnancy; Obstetric history; antenatal care; characteristics of current pregnancy (clinical and obstetric intercurrences, type of fetal presentation, multiple pregnancy, congenital malformations); Care in labour and birth (induction/acceleration of birth, maternal position, venous hydration, use of pain relief medication, anaesthesia, restriction of bed vacancies, presence of a companion during labour, birth and postnatal hospital stay).

### Statistical analyses

Prevalence and respective confidence intervals were estimated for all outcomes in this study taking into consideration the sampling strategy used. The association between women’s demographic and socioeconomic variables and obstetric and neonatal complications were investigated in bivariate, stratified and multivariate model analyses. Statistical tests were applied according to the distribution of data and homogeneity of variances of groups being compared. All analyses were into consideration in the complex study sample design.

## Discussion

Caesarean section rates have systematically increased both in developed and developing countries [31,40-42]. Despite the great variation in the sustained rise in Caesarean section rates in developed and developing countries, it has warranted particular attention in the so-called emerging economies, where Caesarean section rates have accelerated more than in the other countries, reflecting the rapid increase in access to health care services and excessive consumption of medical technology [42-44]. Examples are China [45-47] and Brazil [48], where these increased rates are closely related to the use of private health care services [1,49].

Likewise, Brazilian studies conducted in certain middle and large cities have denounced excessive medicalization in obstetric care for vaginal births such as the routine practice of episiotomy, indiscriminate use of oxytocin in labour and other non-recommended procedures. At the same time, there is low use of continuous labour monitoring, such as use of a partogram, measurement of arterial blood pressure, fetal heartbeats and non-pharmacological pain-relief [1]. Although 98% of births occur in hospitals, cases of difficult access and fragmentation between primary care (antenatal) and hospital (birth) care have been reported [17].

This study, for the first time, depicts the national panorama for labour and birth care in Brazil by geographic region, capital and no capital cities and in the private and public sectors. It also seeks to understand the motivation of women who choose to have Caesarean sections, within a context of a program to prioritize normal labour and birth care, called *Rede Cegonha* (Stork Network), is being launched by the Brazilian government. The purpose of *Rede Cegonha* is to address the challenges caring for birthing women in a hierarchized system and in a humanized way. Countries of continental sizes and with significant social inequality, such as Brazil, have greater difficulties in providing and operating health care services, due to the logistical problems arising from the great distances, access difficulties and inadequate infrastructure in remote areas where a large contingent of the population lives.

This study promotes the development of a network of undergraduate, graduate and post-graduate researchers and students that worked together to collect data throughout Brazil. It collaborated in the training of novice researchers, awakening scientific vocations and providing support to the development of their theses and dissertations.

The results provide inputs towards the implementation of a program to reduce excessive Caesarean sections, which will enable a reduction in maternal and neonatal morbidity/mortality, time in hospital and costs of this care, especially those arising from neonatal ICU admissions. We hope that the results of this study can provide convincing arguments to the Ministry of Health, state and local managers, professional associations and private healthcare plan operators to establish a pact committed to changes in obstetric practices in Brazil, and to implement a evidence-based model of care, which promotes quality, effectiveness, patient safety and respects the rights of women and their families.

These results will be made available to scientific communities, Health System managers and health professionals, as well as to the general population, with particular emphasis on women of reproductive age and their families.

## List of abbreviations

FIOCRUZ: Fundação Oswaldo Cruz (Oswaldo Cruz Foundation); GA: gestational age; ICU: intensive care unit; IMCU: intermediate care unit; LMP: Last menstrual period; SINASC: Sistema de informações de nascidos vivos (Live Birth Information System); US: ultrasound.

## Competing interests

The authors declare that they have no competing interests.

## Authors’ contribution

MCC developed the study, designed and coordinated the research nationally, wrote initial draft of the paper and had primary responsibility for the final content. MABD, AAMS, SGNG, MMTF, RMSMD, JAT, MEM, SDAB, DR, ED, ALC, AJML, RSC, SL and CSGD colaborated in the study design, study coordination and reviewed the final draft of the paper. APEP participated in the sampling process and wrote the final draft of the paper. CLS was responsible for the sampling process and contributed to the writing of the paper. All authors read and approved the final manuscript.

## Financial support

This research received financial support from the National Council for Scientific and Tecnological Development (CNPq) and National School of Public Health - Fiocruz, Brazil.

## Supplementary Material

Additional file 1 FACE-TO-FACE INTERVIEW WITH THE POSTNATAL WOMAN full questionnaire. Click here for file

Additional file 2 Information from woman and newborn’s medical record book full questionnaire. Click here for file

Additional file 3 PHONE INTERVIEW FROM 43 TO 60 DAYS AFTER BIRTH full questionnaire. Click here for file
