# Supplementary material for: Birth in Brazil: national survey into labour and birth
Source: Reprod Health. 2012 Aug 22;9:15. doi: 10.1186/1742-4755-9-15 (PMC3500713; doi:10.1186/1742-4755-9-15)
Supplement: Additional file 1 — FACE-TO-FACE INTERVIEW WITH THE POSTNATAL WOMAN full questionnaire. [file 1742-4755-9-15-S1.pdf]

BIRTH IN BRAZIL - NATIONAL SURVEY INTO LABOUR AND BIRH			
FACE-TO-FACE INTERVIEW WITH THE POSTNATAL WOMAN			
**Part**	**N°**	**QUESTIONS**	**ALTERNATIVES**
**1**	**1**	Time at start of interview	Time format
**1**	**2**	Date of interview	Date format
**1**	**3**	Date of delivery	Date format
**1**	**4**	Medical records number	Number format
**1**	**5**	Type of pregnancy	1. Single 2. Twin (two) 3. Triplets (three) 4. Quadruplets(four)
**1**	**6**	Outcome newborn 1	1. Live 2. Stillbirth 3. Neonatal Death 9. Did not know the answer
**1**	**7**	Name of 1st newborn	String format
**1**	**8**	Outcome newborn 2	1. Live 2. Stillbirth 3. Neonatal Death 9. Did not know the answer
**1**	**9**	Name of 2nd newborn	String format
**1**	**10**	Outcome newborn 3	1. Live 2. Stillbirth 3. Neonatal Death 9. Did not know the answer
**1**	**11**	Name of 3rd newborn	String format
**1**	**12**	Outcome newborn 4	1. Live 2. Stillbirth 3. Neonatal Death 9. Did not know the answer
**1**	**13**	Name of 4th newborn	String format
**2**	**14**	What is your full name?	String format
**2**	**15**	What's your mother's full name?	String format
**2**	**16**	What is your date of birth?	Date format
**2**	**17**	Age (calculated automatically and then confirmed by the mother)	Number format
**2**	**18**	Your skin colour/ ethnicity is ... (Read the alternatives)	1. White 2. Black 3. Brown/ mulatto/ mixed race 4. Yellow /Asian 5. Indigenous
**2**	**19**	Interviewer: In your point of view what skin colour, race or ethnicity the postnatal women belongs to?	1. White 2. Black 3. Brown/ mulatto/ mixed race 4. Yellow /Asian 5. Indiginous
**2**	**20**	What is your address?	String format
**2**	**21**	Landmark to get to the address	String format
**2**	**22**	Phone numbers (with area code)	Number format
**2**	**23**	Residential	Number format

**2**	**24**	Mobile	Number format
**2**	**25**	The husband/ partner or a family member's phone number	Number format
**2**	**26**	Above given telephone owner's name	String format
**2**	**27**	From another relative or neighbour	Number format
**2**	**28**	Above given telephone owner's name	String format
**2**	**29**	Work telephone number (woman's or husband's/partner's)	Number format
**3**	**30**	Before the pregnancy with (name of the baby), how many times have you been pregnant, taking into account any abortion or miscarriages you might have had?	Number format
**3**	**31**	Before the pregnancy with (name of the baby), have you had any abortion or miscarriage before completing 5 months of pregnancy?	0. No (go to 34) 1. Yes
**3**	**32**	How many abortions?	Number format
**3**	**33**	How many of these were spontaneous abortion/miscarriage?	Number format
**3**	**34**	Before the pregnancy with (name of the baby), how many times have you given birth?	(if 00, go to 55)
**3**	**35**	How many of these deliveries were vaginal **(including forceps and vaccum) ?**	Number format
**3**	**36**	How many by caesarean section?	Number format (if no previous caesarean, go to 40)
**3**	**37**	What was the date of your last caesarean section before the birth of (name of the baby)?	Date format

**3**	**38**	What was the reason for the C/S before giving birth to (baby's name)?	01. Wanted to have a tubal ligation<br>02. Had a previous cesarean section<br>03. Did not want to feel the pain of a vaginal birth<br>04. Fear that the hospital beds would be full<br>05. Fear of violence in the city<br>06. The umbilical cord was around the baby's head<br>07. Baby was breech/transverse position<br>08. The baby was large/ I had no dilatation / the baby did not come down/ the baby did not no fit in the pelvis<br>09. Post-maturity/post-date<br>10. The baby was in distress<br>11. Low amniotic fluid volume<br>12. Placenta previa<br>13. High blood pressure<br>14. Diabetes<br>15. HIV / AIDS<br>16. Genital warts / condyloma or problem in the cervical smear for cancer screening exam<br>17. A positive Vaginal swab for Streptococcus<br>18. Placenta abruptio<br>19. Bleeding<br>20. Another reason not mentioned (answer 39)
**3**	**39**	Any other reason not specified above?	String format
**3**	**40**	Before the pregnancy with (name of the baby), how many of your children were born alive?	(if 00, go to 43)
**3**	**41**	Before the pregnancy with (name of the baby), have you given birth to a live baby that died within the first month?	0. No (go to 43) 1. Yes
**3**	**42**	If yes, how many?	Number format
**3**	**43**	Before the pregnancy with (name of the baby), have you given birth to any stillborn at 5 months of gestation or more or weighing more than 500g?	0. No (go to 45) 1. Yes
**3**	**44**	If yes, how many?	Number format
**3**	**45**	Before the pregnancy with (name of the baby), have you given birth to a baby weighing less than 2500g?	0. No (go to 47) 1. Yes
**3**	**46**	If yes, how many?	Number format

**3**	**47**	Before the pregnancy with (name of the baby), have you given birth to a premature baby (before term)?	0. No (go to 49) 1. Yes
**3**	**48**	If yes, how many?	Number format
**3**	**49**	At the other times you were pregnant did you experience any of these events? (Read all options bellow)	-
**3**	**50**	Cerclage/ cervix stitiched to keep baby *in utero*	0. No 1. Yes
**3**	**51**	Eclampsia / convulsions?	0. No 1. Yes
**3**	**52**	High blood pressure, requiring baby to be delivered before term?	0. No 1. Yes
**3**	**53**	Uterine Rupture / the uterus ruptured?	0. No 1. Yes
**3**	**54**	Diabetes / elevated blood sugar?	0. No 1. Yes
**3**	**55**	Have you ever had a surgery on the uterus (i.e. to remove fibroids, mini-cesarean to interrupt pregnancy, surgery to correct infertility, or to treat uterine problems or other causes?)	0. No 1. Yes
**4**	**56**	When you got pregnant, did you (read options):	1. want to get pregnant at the time<br>2. want to wait a bit more<br>3. didn't want to get pregnant
**4**	**57**	How did you feel when you found out you were pregnant with (name of the baby)? (Read options)	1. Satisfied 2. A little satisfied 3. Dissatisfied
**4**	**58**	Did you try interrupting the current pregnancy using any medication or some other method?	0. No (go to 60) 1. Yes
**4**	**59**	If yes, how many weeks of gestation were you when you tried interrupting the current pregnancy?	0. No 1.Yes
**4**	**60**	What was the first day of your last menstrual period (before birth)?	0. No 1. Yes (go to 65)
**4**	**61**	Are you certain of this date?	0. No 1.Yes
**4**	**62**	Did you attend antenatal care during the pregnancy with (name of the baby)?	0. No 1.Yes

**4**	**63**	If not, why didn’t you attend antenatal care? **(do not read the options)**	01. Did not know she was pregnant<br>02. Did not want this pregnancy<br>03. In her oppinion it is not important<br>04. Didn't know it was necessary<br>05. Didn't have money<br>06. Had no one to go with her<br>07. The healthcare service was distant or difficult to access<br>08. Tried but couldnt book the appointment (the service was fully booked)<br>09. Had to wait too long to have the appointment<br>10. I could not attend during the opening hours<br>11. The professional was a male (and she wanted a female)<br>12. She did not like the professionals who worked at that place<br>13. Transportation difficulties<br>14. Another reason (answer the question 64)
**4**	**64**	Any other reason you didn’t attend antenatal care?	(go to 84)
**4**	**65**	How many weeks or months of pregnancy were you when you had the first antenatal care visit/first booking?	-
**4**	**66**	Weeks	Number format
**4**	**67**	Months	Number format
**4**	**68**	**(for those who started after 16 weeks of gestation)** Why didn’t you start antenatal care earlier? **(Do not read the options)**	1. Difficult access (tried but could not get an appointment before)<br>2. Family difficulties (didn`t have anyone to look after other children or no one to go with her)<br>3. Financial difficulties (had no money for transport)<br>4. Personal issues (was not sure she wanted to keep this pregnancy or didn't think it is important to start antenatal care early)<br>5. Difficulties related to work / school (lack of time to attend the appointments)<br>6. Did not know she was pregnant<br>7. Other (answer 69)
**4**	**69**	Any other reason you didn’t start antenatal care earlier?	String format
**4**	**70**	How many antenatal care visits with a doctor, nurse or midwife did you have during the pregnancy with (name of the baby)?	Number format
**4**	**71**	During the pregnancy with (name of the baby) did you receive an antenatal card?	0. No 1. Yes

**4**	**72**	In which sector of healthcare did you have most of the antenatal care visits during the pregnancy with (name of the baby)? **(Read options)**	1. In the public sector 2. In the private sector or covered by medical insurance (go to 74) 3. In both
**4**	**73**	Where (name of the place) did you have these antenatal care visits?	String format
**4**	**74**	What health professional attended you for most antenatal care visits during the pregnancy with (name of the baby)? **(Read options)**	1. Doctor 2. Nurse 3. Midwife 4. Other 9. Does not know
**4**	**75**	During antenatal care were you booked to be seen by the same professional? **(Read options)**	0. No 1. Yes, most of the time 2. Yes, all the time
**4**	**76**	Did you have any ultrasound scan during the pregnancy with (name of the baby)?	0. No (go to 78) 1. Yes
**4**	**77**	How many ultrasounds scans have you had during the pregnancy with (name of the baby)?	Number format
**4**	**78**	During the antenatal care of (name of the baby), were you informed about: (Read options)	-
**4**	**79**	How labour begins?	0. No 1. Yes
**4**	**80**	Danger signs in pregnancy that are alert you to seek *the* healthcare service?	0. No 1. Yes
**4**	**81**	About things you could do during labour to help the birth (i.e. walking, bathing, birthing positions, non-pharmacological ways to reduce pain, etc.)?	0. No 1. Yes
**4**	**82**	Breastfeeding within the first hour of birth?	0. No 1. Yes
**4**	**83**	From what you understood during the antenatal care visits, would you say that in a straightforward pregnancy (without complications): **(Read options)**	1. A vaginal birth is safer for the mother<br>2. A cesarean section is safer for the mother<br>3. Both vaginal birth and cesarean section are safe for the mother<br>4. It was not clear
**4**	**84**	During the pregnancy with (name if the baby) any health professional told you that you had any of the following problems: **(Read options below)**	-

**4**	**85**	The cervix could not hold the baby	0. No 1. Yes
**4**	**86**	Problems with the baby's growth in the womb	0. No 1. Yes
**4**	**87**	Low amniotic fluid volume	0. No 1. Yes (go to 89)
**4**	**88**	High amniotic fluid volume	0. No 1. Yes
**4**	**89**	Rh-negative blood	0. No 1. Yes
**4**	**90**	Low-lying plcenta or placenta praevia	0. No 1. Yes
**4**	**91**	Placenta abruption after the 7th month of pregnancy	0. No 1. Yes
**4**	**92**	Loss of amniotic fluid due to water breaking spontaneously	0. No 1. Yes
**4**	**93**	Gestational Diabetes (elevated blood sugar caused by pregnancy)	0. No 1. Yes
**4**	**94**	Gestational hypertention (High blood pressure caused by pregnancy)	0. No 1. Yes
**4**	**95**	Eclampsia / Convulsions	0. No 1. Yes
**4**	**96**	Threat of premature labour	0. No 1. Yes
**4**	**97**	Signs of baby distress	0. No 1. Yes
**4**	**98**	Syphilis	0. No 1. Yes
**4**	**99**	Urinary tract infection / cystitis	0. No 1. Yes
**4**	**100**	HIV / AIDS	0. No 1. Yes
**4**	**101**	Toxoplasmosis (that needed to be treated)	0. No 1. Yes
**4**	**102**	Positive culture for streptococcus in the vagina	0. No 1. Yes
**4**	**103**	Other infectious diseases	0. No (go to 105) 1. Yes
**4**	**104**	Which other infectious diseases?	String format
**4**	**105**	Other problems	0. No (go to 107) 1. Yes
**4**	**106**	Which other problems?	String format
**4**	**107**	Were you considered to have a high-risk pregnancy?	0. No (go to 110) 1. Yes
**4**	**108**	Were you sent to another hospital because you were a high-risk pregnancy?	0. No (go to 110) 1. Yes
**4**	**109**	Were you admmited to give birth in the same hospital that provided you with the antenatal care? **(Read options)**	0. No 1. Yes, but it was difficult 2. Yes, without any difficulties

**4**	**110**	During the pregnancy with (name of the baby) were you ever hospitalized?	0. No (go to 113) 1. Yes
**4**	**111**	If yes, what was the reason given? **(Do not read the options)**	01. Hypertension / preeclampsia<br>02. Bleeding<br>03. Threat of preterm birth<br>04. Excessive vomiting<br>05. Diabetes<br>06. Was losing water<br>07. Urinary tract infection<br>08. Low amniotic liquid/ high amniotic liquid<br>09. Other (answer 112)
**4**	**112**	Other reason not specified above? Which reason?	String format
**4**	**113**	During the pregnancy with (name of the baby), were you instructed to which hospital / maternity unit / birth center to seek care when in labour?	0. No (go to o part V) 1. Yes
**4**	**114**	Did you give birth in the place you were instructed to seek?	0. No 1. Yes (go to o part V)
**4**	**115**	If not, why? **(Do not read options)**	1. It was full<br>2. It was far away or difficult to get there<br>3. She doesn't like the care provided at this place<br>4. Other (answer 116)
**4**	**116**	If another reason not specified above, describe.	String format
**5**	**117**	At the beginning of pregnancy with (name of the baby), what type of birth/mode of delivery did you prefer? **(Read options)**	1. Vaginal birth<br>2. Caesarean delivery<br>3. Had no preference at all (go to 120)

**5**	**118**	In your oppinion what might have influenced your preference, in early pregnancy, in relation to type of birth/mode of delivery? **(Do not read the options)**	01. Stories of births in her family and /or her friends<br>02. The husband's preference for type of birth/mode of delivery<br>03. Fear of vaginal birth<br>04. Fear that vaginal birth would alter her vagina<br>05. She wanted to have a tubal ligation<br>06. The fear of caesarean section<br>07. Fear of the anaesthesia<br>08. To schedule the date for delivery<br>09. To have known the healthcare professional that would assist the birth<br>10. Previous positive experience with vaginal birth<br>11. Previous negative experience with vaginal birth<br>12. Previous positive experience with caesarean<br>13. Previous negative experience with caesarean<br>14. Information found on the Internet<br>15. Information found in newspapers and magazines<br>16. Information found on television<br>17. Information found on discussion groups of pregnant women (childbirth preparation groups)<br>18. Vaginal birth is better than cesarean section<br>19. Better recovery in vaginal birth<br>20. Other (answer 119)
**5**	**119**	In case she reported other reason not included in the options above, describe the reasons here.	String format
**5**	**120**	At the end of your pregnancy with (name of the baby), near the date of the birth, had the type of delivery already been decided?	0. No (go to part VI)<br>1. Yes, vaginal birth<br>2. Yes, caesarean section
**5**	**121**	If yes, who made this decision? **(Read options)**	1. You<br>2. The doctor<br>3. Joint decision<br>4. Other person (answer the question 122)
**5**	**122**	If another person not specified above, describe here.	String format

**6**	**123**	What made you think that it was time to seek a hospital for giving birth to (name of the baby)? **(Do not read options)**	01. She was going into labour<br>02. The waters broke<br>03. Had the show / loss of cervical mucus plug<br>04. She was in pain / had contractions<br>05. The date of her C-section was scheduled<br>06. She was referred from antenatal care<br>07. For induction of labour<br>08. She was sick (high blood pressure, bleeding, etc ...)<br>09. It passin the baby's due date<br>10. The baby was not moving<br>11. The baby was in distress<br>12. Other (answer the question 124)
**6**	**124**	If another reason not specified above, describe here.	String format
**6**	**125**	Before being admitted to this hospital / maternity unit did you seek another hospital / maternity unit?	0. No (go to 129) 1. Yes
**6**	**126**	If yes, how many?	Number format
**6**	**127**	Why were you not admitted in the hospital(s)/maternity unit(s) you first sought? **(Do not read options)**	1. It was full/no vaccant beds<br>2. She was not in labour<br>3. They sent her to another hospital for high risk pregnancies (High dependency unit)<br>4. There were no doctors in the shift at the hospital/ the hospital didn't have resources/conditions to provide her care<br>5. She was not informed<br>6. Other (answer the question 128)
**6**	**128**	If another reason not specified above, describe here.	String format
**6**	**129**	How did you get to the hospital/ maternity unit? **(Read options)**	1. Walking 2. Car 3. Public transport 4. Taxi 5. Ambulance<br>6. Other (answer the question 130)
**6**	**130**	If in a different transport not specified above, describe here.	String format
**6**	**131**	How long did it take you to arrive in this hospital / maternity unit / birth center where you have given birth?	Number format
**6**	**132**	Once you arrived at the hospital / maternity unit / birth center, how long did it take for you to be admitted?	Number format
**6**	**133**	Did you have a vaginal examination when you were admitted to the hospital?	0. No (go to 135) 1. Yes
**6**	**134**	How many centimeters dilatated were you at the time of admission?	Number format 000. No dilatation
**6**	**135**	Did anyone hear the baby's heart beat at the time of admission?	0. No 1. Yes

**7**	**136**	Did you go into labour? **(Read options)**	0. No (go to 151) 1. Yes (spontaneous or induced)<br>2. No, despite being induced
**7**	**137**	Were you offered liquids, water, juice and / or soup / food during labour?	0. No 1.Yes
**7**	**138**	Have you asked for any liquid or food during labour?	0. No (go to 140) 1.Yes
**7**	**139**	Was your request granted?	0. No 1. Yes
**7**	**140**	When you were in labour, did you have a catheter/cannula in your vein?	0. No (go to 143) 1. Yes
**7**	**141**	Was a medication to increase contractions (oxytocin) added?	0. No (go to 143) 1. Yes 9. Didn't know (go to 143)
**7**	**142**	After this medication was added, did the contractions (labour pain) increase? **(Read options)**	1. You didn't notice the diference<br>2. They increased a little<br>3. They increased very much
**7**	**143**	When you were in labour, was a medication inserted into your vagina to induce/ augment the delivery process?	0. No (go to 145) 1. Yes 9. Didn't know (go to 145)
**7**	**144**	After this medication was put into the vagina, did the contractions (labour pain) increase? **(Read options)**	1. You didn't notice the diference<br>2. They increased a little<br>3. They increased very much
**7**	**145**	Did anyone break your waters after you arrived at this hospital? **(read options)**	1. No, it broke before admission<br>2. No, it broke by itself during your stay at the hospital<br>3. Yes
**7**	**146**	What was the colour of the liquid? **(Read options)**	1. Transparent<br>2. Green/ brown<br>3. With blood<br>4. Yellow/ purulent<br>9. Didn't know
**7**	**147**	Were you allowed to go out of the bed, move around and walk during labour? **(Read options)**	0. No, it was not allowed<br>1. No, but you didn't want to<br>2. Yes
**7**	**148**	Did you do any of the following strategies to relieve pain during labour? **(Read options)**	0. None<br>1. Bath<br>2. Shower<br>3. Birthing ball<br>4. Massage<br>5. Squatting position<br>6. Rocking/birth chair<br>7. Other (answer 149)

**7**	**149**	If one not specified above, describe here.	String format
**7**	**150**	After admission in this hospital/maternity unit, did you have an exam called CTG (exam with two waist bands around your belly to check contractions and the baby's heart beat)?	0. No<br>1. Yes, when you were admitted<br>2. Yes, sometimes during labour<br>3. Yes, throughout all labour<br>9. You don't know
**7**	**151**	Did someone stay with you whilst in hospital/maternity unit stay?	0. No 1. Yes (go to 154)
**7**	**152**	If not, why? **(Do not read options)**<br>**(After this question, go to part VIII)**	01. The hospital did not allow any companion<br>02. No men were allowed as a companion<br>03. Only allowed companion for teenage mothers<br>04. Only allowed an adult companion<br>05. She didn't know she was allowed to have a companion<br>06. She did not want a companion<br>07. She didn't have anyone to stay with her<br>08. Would have to pay to hire a companion<br>09. The hospital only allowed companion at the delivery room<br>10. Other (answer the question 153)
**7**	**153**	If another reason not specified above, describe here.	String format
**7**	**154**	Did the person accompaning you stay with you: **(read options below)**	-
**7**	**155**	During the hospital admission process (before being admitted)?	0. No 1. Yes
**7**	**156**	All the time during labour (before birth)?	0. No 1. Yes 2. Didnt go into labor
**7**	**157**	During birth?	0. No 1. Yes
**7**	**158**	In the immediate postnatal period (on the obstetric ward / recovery room)?	0. No 1. Yes
**7**	**159**	During the hospital stay after delivery (stayed with you in the room / ward)?	0. No 1. Yes
**7**	**160**	Who stayed with you? (check more than one if applicable)	1. Partner or child`s father<br>2. A Friend<br>3. Mother<br>4. Sister<br>5. Doula<br>6. Other peolpe? (answer the question 161)
**7**	**161**	If anyone not included in the alternatives above, describe here.	String format

**7**	**162**	The person that stayed with you was the one that you had previously chosen?	0. No 1. Yes
**7**	**163**	How would you describe the experience of having a person with you as a companion during labour? **(read options)**	1. It helps very much the woman to stay calm and have a better birth<br>2. It helps a little the woman to stay calm and have a better birth<br>3. Neither helps nor hinders the woman to stay calm and have a better birth<br>4. Makes the woman more nervous, does not help to have a better birth
**7**	**164**	With how many weeks of gestation or months of pregnancy was the baby born?	-
**8**	**165**	Weeks	Number format
**8**	**166**	Months	Number format
**8**	**167**	During pregnancy with (name of the baby) did you have an injection (of steroids) to mature the baby's lung? **(read options)**	0. No (go to 171)<br>1. Yes during antenatal care<br>2. Yes, at the hospital during a previous admission<br>3. Yes, at the hospital, is this admission
**8**	**168**	Do you remember how many weeks / months of pregnancy you were when you had this injection? **(if reported in weeks, do not score months)**	00. No 1. Yes, how many months/ weeks
**8**	**169**	Weeks	Number format
**8**	**170**	Months	Number format
**8**	**171**	The person who assisted your labour/ birth was the same who attended you during atenatal visits?	0. No 1. Yes
**8**	**172**	What was the type of birth? **(read options)**	1. Vaginal birth<br>2. Vaginal birth with forceps<br>3. Caesarean section (go to 181)<br>(if twins/multiples, inform the type of birth for all)
**8**	**173**	What professional assisted your labour and birth? **(do not read options)**	1. Doctor<br>2. Nurse<br>3. Midwife<br>4. Student<br>5. Nobody appeared<br>6. Gave birth alone<br>7. Other (answer to question 174)
**8**	**174**	If another professional/ roles not specified above, describe here.	String format
**8**	**175**	During labour, did you have to move to another room when it was time to push to give birth?	0. No 1.Yes

**8**	**176**	In which position did you have the baby? **(read options)**	1. Lying on your back with legs raised<br>2. Lying on one side<br>3. Sitting / reclining<br>4. in the bathtub<br>5. All fours support<br>6. squatting<br>7. standing up
**8**	**177**	At the time of birthing the baby, did someone instruct you to push or put pressure on your belly to help the baby out? (Kristeller manouvre)	0. No 1. Yes
**8**	**178**	Do you know how was your perineum (the vagina) after birth? **(read options)**	1. There were no lacerations, cut or sutures<br>2. A little laceration, but did not need stitches<br>3. You don't have stitches, but don't know if there is any laceration<br>4. There was a laceration and you have had stitches<br>5. They cut and you had stitches<br>6. You have stitches, but don't know if there was laceration or if they cut<br>(If you answer 1, 2 or 3, go to 180)
**8**	**179**	Was your perineum under local anesthesia before the episiotomy (being cut) or before suturing? **(Read options)**	0. No 1. Yes, before being cut (episiotomy) 2. Yes, before the stitches 9. didn't know the answer
**8**	**180**	Was any anesthesia/analgesia applied on your back anytime during the labour or birth? **(Read options)**	0. No 1.Yes, during labour 2. Yes, at birth 9. Didn't know the answer
**8**	**181**	At the time of birth in which position was the baby in your belly? **(Read options)**	1. Vertex (head first position) 2.Breech 3. Other position
**8**	**182**	Position of the second baby	1. Vertex (head first position) 2.Breech 3. Other position
**8**	**183**	Position of the third baby	1. Vertex (head first position) 2.Breech 3. Other position
**8**	**184**	Position of the fourth baby	1. Vertex (head first position) 2.Breech 3. Other position
**8**	**185**	At what point it was decided that a caesarean section was necessary? **(Read options)**	1. During antenatal care<br>2. During stay is hospital as a pregnant woman<br>3. At admission to give birth<br>4. In the labour ward<br>5. In the delivery room

**8**	**186**	What was the reason given for the caesarean section? **(Do not read options)**	01. Wanted a caesarean<br>02. Wanted to have a tubal ligation<br>03. The umbilical cord was around the baby's head<br>04. Had a previous caesarean section<br>05. Had two or more previous caesarean sections<br>06. Breech position<br>07. Transverse position<br>08. The baby was big/ had no dilatation / the baby`s head did not fit or accomodated to the pelvis<br>09. Low amniotic fluid volume / old placenta<br>10. Did not want to feel the pain of vaginal birth<br>11. The baby had a restricted growth or stopped growing<br>12. The baby was in distress<br>13. Post-maturity<br>14. The waters broke<br>15. Pregnant with twins (or multiples)<br>16. High blood pressure<br>17. Bleeding<br>18. Diabetes<br>19. Fear that all hospital beds would be occupied<br>20. Fear of violence in the city<br>21. Stillbirth<br>22. Previous gynaecologic surgery (perineoplasty, myomectomy micro-caesarian)<br>23. Placenta previa<br>24. Failed induction / induction did not work<br>25. Another reason not mentioned (answer the question 187)
**8**	**187**	If another reason not specified above, describe here.	String format
**8**	**188**	Did the baby poo (meconium) while still in the womb?	0. No 1.Yes 9. Didn't know the answer
**9**	**189**	Shortly after giving birth, while in the delivery room before the first weighing, measuring, etc of the baby, you: **(Read options)**	1. Offered your breasts 2. Held the baby<br>3. Just saw the baby 4. Didn`t have any contact with the baby
**9**	**190**	Did the baby came into the postnatal ward with you?	0. No 1. Yes (go to 197)
**9**	**191**	If not, why? **(Read options)**	1. The baby was sent to nursery/ warm cradle/ incubator<br>2. The baby was sent to intermediate or intensive care unit<br>3. Other reason (answer question 192)
**9**	**192**	If another reason not specified above, describe here.	String format
**9**	**193**	How long (days, hours or minutes) after birth did your baby come to stay with you in your room?	Number format
**9**	**194**	Days	Number format

**9**	**195**	Hours	Number format
**9**	**196**	Minutes	Number format
**9**	**197**	Did your baby have any of these problems or needs? (Read options)	-
**9**	**198**	Hypoglycemia - low blood sugar	0. No 1.Yes
**9**	**199**	Congenital malformation (including congenital heart defect)	0. No 1.Yes
**9**	**200**	Needed oxygen after birth	0. No 1.Yes
**9**	**201**	Turned yellow (jaundice)	0. No 1.Yes
**9**	**202**	Bathed in light	0. No 1.Yes
**9**	**203**	Transferred to another hospital	0. No 1.Yes
**9**	**204**	Infection	0. No 1.Yes
**9**	**205**	Other problem/ needs	0. No (go to 207) 1.Yes
**9**	**206**	Descibe the other problem here.	String format
**9**	**207**	Have you offered you breasts to your baby yet?	0. No (go to 213) 1. Yes 8. Not applicable
**10**	**208**	After birth, did you offer your breasts in the delivery room?	0. No 1. Yes (go to 215)
**10**	**209**	How long did it take for you to offer your breasts to your baby for the first time? (More or less)	-
**10**	**210**	Days	Number format
**10**	**211**	Hours	Number format
**10**	**212**	Minutes	Number format
**10**	**213**	Why haven`t you offered your breast to your baby yet? **(do not read options)**	1. Mother is HIV+<br>2. Mother HTLV+<br>3. Premature baby<br>4. The baby was sick or could not be breastfed<br>5. Didn`t have enough milk<br>6. Didn`t have a comfortable position to breastfeed yet<br>7. Others (answer question 214)
**10**	**214**	If another reason not specified above, describe here.	String format

**10**	**215**	(Here) in this hospital, has your baby had any milk or other liquids other than your breast milk?	0. No (if twins or multplies, go to part 11, if singleton, go to part 17)<br>1. Yes<br>8. Not applicable (if twins or multiples, go to part 11, if singleton, go to part 17)<br>9. Does nor know (if twins or multiples, go to part 11, if singleton, go to part 17)
**10**	**216**	Why did the baby have other milk or liquids? **(Do not read options)**	1. Premature Baby<br>2. The baby was sick<br>3. Didn`t have enough milk<br>4. Routine of the hospital<br>5. Didn`t have a comfortable position to breastfeed yet<br>6. It was prescribed by the pediatrician<br>7. Other (answer 217)<br>9. Didn `t know the answer
**10**	**217**	If another reason not specified above, describe here.	String format
**10**	**218**	How was the milk / liquid given to your baby? **(Read options)**	1. In the bottle<br>2. in the cup<br>3. In the probe / gavage / syringe<br>4. Other (answer 219)<br>9. Didn `t know the answer
**10**	**219**	If in another way not specified above, describe here.	String format
**QUESTIONS 220 TO 315 (PARTS 11 TO 16) ARE RELATED TO TWINS. THE QUESTIONS ARE IDENTICAL TO QUESTIONS 189 TO 219 (PARTS 9 AND 10)**			
**17**	**316**	Are you able to read and write?	0. No 1. Yes
**17**	**317**	What was the highest level of education you have entered?	0. None (go to 319)<br>1. Primary school<br>2. Secondary school<br>3. University<br>9. Didn`t know the answer
**17**	**318**	What was the last grade you completed in this stage?	Number format

**17**	**319**	What is your marital status? (Read options)	1. Single<br>2. Married<br>3. in a stable relationship/ live with partner<br>4. Separated/ divorced<br>5. Widow
**17**	**320**	Do you work (and get paid for that)?	0. No (go to 323) 1. Yes
**17**	**321**	In relation to your work, you: **(read options)**	01. Formally employed<br>02. Work informally<br>03. Work for the government<br>04. Employer<br>05. Autonomous<br>06. Cooperative<br>07. Other (answer the question 322)
**17**	**322**	If another work/employment situation not specified above, describe here.	String format
**17**	**323**	Who is (the) head of the family? **(do not read options)**	1. She (go to part XVIII)<br>2. Her partner/husband<br>3. Her mother<br>4. Her father<br>5. Other family member (answer 324)<br>6. Other person that doesn`t live in the same household (answer 324)
**17**	**324**	If another person not specified above, describe here.	String format
**17**	**325**	Which was the highest level of education of the head of the family?	0. None (go to 329)<br>1. Primary school (go to 326)<br>2. Secondary school (go to 327)<br>3. University (go to 328)<br>9. Didn`t know the answer
**17**	**326**	What was the last grade the head of the family completed at primary school?	Number format
**17**	**327**	What was the last grade the head of the family completed at secondary school?	Number format
**17**	**328**	What was the last year the head of the family completed at university?	Number format
**17**	**329**	How many people live in the same household, including yourself? (do no count the newborn(s))	Number format

**18**	**330**	How many rooms (including living rooms) trere are in your house?	Number format
**18**	**331**	Do you have a bathroom at your house for an exclusive use of your family?	0. No (go to a 333) 1. Yes
**18**	**332**	How many bathrooms in your house (inside or outside) has a toilet?	Number format
**18**	**333**	Now, I will ask you some questions about things you may or may not have in your house.	-
**18**	**334**	Radio	0. No (go to 336) 1. Yes
**18**	**335**	How many?	1. One 2. Two 3. Three 4. More than three
**18**	**336**	Refrigerator	0. No 1. Yes
**18**	**337**	Freezer (independent device or part of refrigerator duplex)	0. No 1. Yes
**18**	**338**	DVD or VCR	0. No 1. Yes
**18**	**339**	washing machine	0. No 1. Yes
**18**	**340**	Colour television	0. No (go to 342) 1. Yes
**18**	**341**	How many?	1. One 2. Two 3. Three 4. More than three
**18**	**342**	Motorcycle	0. No 1. Yes
**18**	**343**	Car (for private use)	0. No (go to 345) 1. Yes
**18**	**344**	How many?	1. One 2. Two 3. Three 4. More than three
**18**	**345**	In your home has housemaids? (5 or more days per week)	0. No (go to 347) 1. Yes
**18**	**346**	How many?	1. One 2. More than one
**19**	**347**	Did you smoke before pregnancy?	0. No 1. Yes
**19**	**348**	Did you smoke during the first five months of pregnancy?	0. No (go to 351) 1. Yes
**19**	**349**	During the first five months of pregnancy did you use to smoke every day?	0. No 1. Yes
**19**	**350**	During the first five months of pregnancy how many cigaretts did you use to smoke per day?	Number format
**19**	**351**	Did you smoke after the fifth month of pregnancy?	0. No (go to 354) 1. Yes
**19**	**352**	After the fifth month of pregnancy did you use to smoke every day?	0. No 1. Yes
**19**	**353**	After the fifth month of pregnancy how many cigaretts did you use to smoke per day?	Number format

**19**	**354**	During pregnancy, did you drink beer or other alcoholic beverage?	0. No<br>1. Yes<br>If the woman didn`t drink any alcohol during pregnancy go to part 20
**19**	**355**	Have you ever felt you should Cut down on your drinking? **(CAGE & T-ACE)**	0. No 1. Yes
**19**	**356**	Does your husband (or parents) ever worry or complain about your drinking? / Have people Annoyed you by criticizing your drinking? **(CAGE & T-ACE)**	0. No 1. Yes
**19**	**357**	Have you ever had a drink first thing in the morning to steady your nerves or to get rid of a hangover? **(Eye opener) (CAGE & T-ACE)**	0. No 1. Yes
**19**	**358**	Have you ever awakened in the morning after some drinking the night before and found that you could not remember a part of the evening before?	0. No 1. Yes
**19**	**359**	Does it take more than three drinks to make you feel high? **(T-ACE)**	0. No 1. Yes
**19**	**360**	Did you have some of these diseases before pregnancy was confirmed by a doctor? **(Read options below)**	-
**20**	**361**	Heart disease	0. No 1. Yes
**20**	**362**	Non-gestational High blood pressure with prescribed medication for continued use	0. No 1. Yes
**20**	**363**	Severe anemia, not during pregnancy, or other blood disorder	0. No 1. Yes
**20**	**364**	Asthma / bronchitis	0. No 1. Yes
**20**	**365**	Lupus or scleroderma	0. No 1. Yes
**20**	**366**	Hyperthyroidism	0. No 1. Yes
**20**	**367**	Non-gestational Diabetes / high blood sugar, confirmed by medical specialist	0. No 1. Yes
**20**	**368**	Kidney disease / kidney confirmed by medical specialist who needs treatment	0. No 1. Yes
**20**	**369**	Epilepsy / seizure before pregnancy	0. No 1. Yes
**20**	**370**	CVA / stroke	0. No 1. Yes
**20**	**371**	Liver disease confirmed by medical specialist who needs treatment	0. No 1. Yes
**20**	**372**	Mental illness, which requires monitoring by a specialist	0. No 1. Yes

**20**	**373**	Other condition	0. No (go to 375) 1. Yes
**20**	**374**	Describe other condition	String format
**21**	**375**	Do you have a private medical insurance? **(Read options)**	0. No (go to part 22)<br>1. Yes, one<br>2. Yes, more than one
**21**	**376**	For how long have you had this private medical insurance? **(read options)**	1. Less than 6 months<br>2. From 6 months to 1 year<br>3. From 1 year to 2 years<br>4. More than 2 years<br>9. Didnt know the answer
**21**	**377**	This is an individual or family private medical insurance?	1. Individual (go to 379) 2. Familiar
**21**	**378**	How many people are covered by this insurance?	Number format
**21**	**379**	Who is in charge of paying the costs of this private medical insurance? **(read options)**	1. The company/ the employer (go to 381)<br>2. The employee, debited from the salary<br>3. The holder of the insurance<br>9. Didnt know the answer (go to 381)
**21**	**380**	How much money does it cost every month? (if you have more than one medical insurance, consider the main insurance) **(read options)**	01. <= 30 Reals<br>02. >30 <=50 Reals<br>03. > 50 <= 100 Reals<br>04. > 100 <= 200 Reals<br>05. > 200 <=300 Reals<br>06. > 300 <= 500 Reals<br>07. > 500 Reals<br>99. Didnt know the answer
**21**	**381**	In addition to the montly costs, this medical insurance charges any extra fees for the appointments you have?	0. No 1. Yes 9. Didn`t know the answer
**21**	**382**	This private medical insurance includes medical consultations?	0. No 1. Yes 9. Didn`t know the answer
**21**	**383**	This private medical insurance includes hospital admission?	0. No 1. Yes 9. Didn`t know the answer
**21**	**384**	This private medical insurance includes maternity coverage - care during labour and birth?	0. No 1. Yes 9. Didn`t know the answer
**21**	**385**	This private medical insurance includes having health exams?	0. No 1. Yes 9. Didn`t know the answer

**21**	**386**	Was the care received in the current pregnancy, labour and birth covered by the private medical insurance? **(read options)**	1. Yes, everything<br>2. Yes, only for the prenatal visits<br>3. Yes, only for birth<br>4. Yes, only for the medical exams<br>5. No (answer question 387)<br>9. Didn`t know the answer
**21**	**387**	If not, why?	String format
**22**	**388**	How much did you weigh before pregnancy? (in kg)	Number format
**22**	**389**	How much did you weigh at your last antenatal care visit? (in kg)	Number format
**22**	**390**	When were you last weighed?	date format
**22**	**391**	How tall are you? (in cm)	Number format
**23**	**392**	Would you like to say anything else?	0. No (go to 394) 1. Yes
**23**	**393**	Write down here what she says	String format
**23**	**394**	End time of interview	Time format
**23**	**395**	Antenatal card was photographed?	0. No 1. Yes
**23**	**396**	Observations of the interviewer:	String format
**23**	**397**	Did the woman refuse to be contacted by telephone 42 days after birth?	0. No 1. Yes
