# Supplementary material for: Birth in Brazil: national survey into labour and birth
Source: Reprod Health. 2012 Aug 22;9:15. doi: 10.1186/1742-4755-9-15 (PMC3500713; doi:10.1186/1742-4755-9-15)
Supplement: Additional file 2 — Information from woman and newborn’s medical record book full questionnaire. [file 1742-4755-9-15-S2.pdf]

BIRTH IN BRAZIL - NATIONAL SURVEY INTO LABOUR AND BIRH			
Information from woman and newborn`s medical record book			
Part	Nº	Questions	Alternatives
Part 1		GENERAL DATA	
1	1	Date of data collection from medical record	Date format
1	2	Time of data collection from medical record	Time format
1	3	Name of pregnant/ post-partum woman	String format
1	4	Woman`s number in medical record	Number format
1	5	Type of Pregnancy	1. Single 2. Twins (two) 3. Twins (three) 4. Twins (four)
1	6	Outcome newborn 1	1. Live 2. Stillbirth 3. Neonatal Death
1	7	Outcome newborn 2	1. Live 2. Stillbirth 3. Neonatal Death
1	8	Outcome newborn 3	1. Live 2. Stillbirth 3. Neonatal Death
1	9	Outcome newborn 4	1. Live 2. Stillbirth 3. Neonatal Death
Part 2		INFORMATION ON ADMISSION	
2	10	Date of admission	Date format
2	11	Time of admission	Time format
2	12	Sector where pregnant woman was sent at the time of admission	1. Ward / room 2.Labour ward 3. PPP 4. Delivery room<br>5. Obstetric Surgical Center 6. ICU 9. No Information
2	13	Destination from hospital where the woman gave birth	1. Discharged home/community from hospital<br>2. Transferred in the postpartum period (go to question 15)<br>3. Left hospital without medical autorization<br>4. Death<br>5. Remained hospitalized after 42 days of birth (go to 17)
2	14	Date of discharge from hospital	Date format
2	15	Hospital where woman was transferred after birth (hospital name - city - state)	String format
2	15.1	Reason for being transferred to another hospital	String format
2	15.2	Destination from hospital where women was transferred to	1. Discharged home/community from hospital<br>2. Left hospital without medical autorization<br>3. Death<br>4. Remained hospitalized after 42 days of birth (go to 17)
2	15.3	Date of discharge from hospital where woman was transferred to	Date format
2	16	Death Certificate registry number	Number format
Part 3		CLINICAL-OBSTETRIC HISTORY	

**3**	**17**	Number of previous pregnancies	Number format (If the first pregnancy, complete with 00 and go to question 21)
**3**	**18**	Number of previous miscarriages	Number format
**3**	**19**	Total number of previous deliveries	Number format (if 00, go to question 21)
**3**	**20**	How many deliveries by caesarean section?	Number format
**3**	**21**	**Personal medical history**	-
**3**	**22**	Heart disease	0. No 1. Yes
**3**	**23**	High blood pressure with continued treatment	0. No 1. Yes
**3**	**24**	Severe anemia or other hemoglobinopathy	0. No 1. Yes
**3**	**25**	Asthma	0. No 1. Yes
**3**	**26**	Lupus or scleroderma	0. No 1. Yes
**3**	**27**	Hyperthyroidism	0. No 1. Yes
**3**	**28**	Diabetes (non gestacional)	0. No 1. Yes
**3**	**29**	Chronic kidney disease	0. No 1. Yes
**3**	**30**	Seizures / epilepsy	0. No 1. Yes
**3**	**31**	Cerebral Vascular Accident (Stroke)	0. No 1. Yes
**3**	**32**	Chronic liver disease	0. No 1. Yes
**3**	**33**	Psychiatric illness	0. No 1. Yes
**3**	**34**	Other	0. No (go to 36) 1. Yes
**3**	**35**	Specify others	String format
**3**	**36**	**Obstetric or medical complications in the current pregnancy (before hospital admission)**	-
**3**	**37**	Cervical incompetence (CI)?	0. No 1. Yes
**3**	**38**	Intra Uterine Growth Restriction (IUGR)?	0. No 1. Yes
**3**	**39**	Oligohydramnios?	0. No 1. Yes
**3**	**40**	Polyhydramnios?	0. No 1. Yes
**3**	**41**	RH isoimmunization?	0. No 1. Yes
**3**	**42**	Placenta previa?	0. No 1. Yes
**3**	**43**	Placenta abruption?	0. No 1. Yes
**3**	**44**	Premature rupture of membranes?	0. No 1. Yes
**3**	**45**	Gestational Diabetes?	0. No 1. Yes
**3**	**46**	Hypertensive disorders (chronic hypertension, preeclampsia, HELLP syndrome)?	0. No 1. Yes
**3**	**47**	Eclampsia / Seizures?	0. No 1. Yes
**3**	**48**	Threat of premature labour?	0. No 1. Yes

**3**	**49**	Fetal distress?	0. No 1. Yes
**3**	**50**	Syphilis?	0. No 1. Yes
**3**	**51**	Urinary tract infection?	0. No 1. Yes
**3**	**52**	HIV infection?	0. No 1. Yes
**3**	**53**	Toxoplasmosis (that needed to be treated)?	0. No 1. Yes
**3**	**54**	Positive culture for streptococcus in the vagina?	0. No 1. Yes
**3**	**55**	Birth defects?	0. No (go to 57) 1. Yes
**3**	**56**	Which birth defects?	String format
**3**	**57**	Other problems	0. No (go to 59) 1. Yes
**3**	**58**	Which other problems?	String format
**3**	**59**	Previous surgery on the uterus (i.e. to remove fibroids, micro-caesarean to interrupt pregnancy, or other surgical procedures on the uterus)?	0. No 1. Yes
**Part 4**		**INFORMATION ON ADMISSION**	
**4**	**60**	Date of last menstrual period (LMP):	Date format
**4**	**61**	Gestational age (on admission) calculated by LMP (in weeks):	Number format
**4**	**62**	Gestational age (on admission) measured by previous ultrasound scan (in weeks):	Number format
**4**	**63**	Gestational age (on admission) but method of calculation is not specified (in weeks):	Number format
**4**	**64**	**Baby`s presentation:**	**-**
	**65**	First Baby	1. Vertex (head first) 2.Breech 3. Other position 9. Not registered
**4**	**66**	Second baby	1. Vertex (head first) 2.Breech 3. Other position 9. Not registered
**4**	**67**	Third baby	1. Vertex (head first) 2.Breech 3. Other position 9. Not registered
**4**	**68**	Fourth baby	1. Vertex (head first) 2.Breech 3. Other position 9. Not registered
**4**	**69**	Level of conscious state of woman:	1. Lucid 2. Numbness (confusion)<br>3. In a coma 9. Not registered
**4**	**70**	Occurrence of convulsions before hospital admission?	0. No 1. Yes
**4**	**71**	Any record of blood pressure assessment upon admission?	0. No (go to 74) 1. Yes
**4**	**72**	First check of blood pressure: syst (mmHg):	Number format
**4**	**73**	First check of blood pressure: diast (in mmHg):	Number format
**4**	**74**	Any record of axillary temperature assessment on admission?	0. No (go to 76) 1. Yes

**4**	**75**	Temperature in centigrade	Number format
**4**	**76**	Vaginal bleeding after hospital admission and before delivery?	0. No<br>1. Yes, small<br>2. Yes, moderate<br>3. Yes, intense<br>4. Yes, unspecified
**4**	**77**	Loss of amniotic fluid (rupture of membranes) before hospital admission:	1. No<br>2. Yes, clear liquid with no lumps<br>3. Yes, clear liquid with lumps<br>4. Yes, fluid with meconium<br>5. Yes, bloody fluid<br>6. Yes, purulent fluid / foul<br>7. Yes, unspecified
**4**	**78**	Dilatation of the cervix on admission in centimeters:	Number format
**4**	**79**	Number of contractions every 10 minutes on admission to hospital:	Number format
**4**	**80**	Fetal heart rate (FHR) assessment on admission (or the first examination):	0. Absent (go to 82) 1. Present
**4**	**81**	Frequency of FHR?	Number format
**4**	**82**	Any cardiotocography (CTG)?<br>**it is possible to have more than one answer**	0. No (go to 84)<br>1. Yes, before arriving in hospital<br>2. Yes, on admission<br>3. Yes, during labour
**4**	**83**	Any alteration in CTG?	0. No 1. Yes 9. not registered
**4**	**84**	Any Fetal Doppler flowmetry?<br>**it is possible to have more than one answer**	0. No (go to 86)<br>1. Yes, before arriving in hospital<br>2. Yes, on admission<br>3. Yes, during labour
**4**	**85**	Any alteration in Doppler flowmetry?	0. No 1. Yes 9. not registered
**4**	**86**	Use of corticosteroids before delivery<br>**it is possible to have more than one answer**	0. No (go to 86)<br>1. Yes, before arriving in hospital<br>2. Yes, on admission
**4**	**87**	Reason for going into hospital	1. Spontaneous labour<br>2. Induction of labour<br>3. Elective caesarean section (answer 88 and then go to 130)<br>4. Admission as a pregnant woman, for clinical or obstetric complications<br>5. Another reason

4	88	Diagnosis on hospital admission: (it is possible to have more than one answer)	1. Labour<br>2. Preterm labour / threat of premature labour<br>3. Ruptured membranes<br>4. Multiple pregnancy (two fetuses or more)<br>5. Prolonged pregnancy / post-maturity<br>6. Fetal distress (acute / chronic) - growth restriction (IUGR)<br>7. Polyhydramnios / Oligohydramnios<br>8. Placental abruption<br>9. Vaginal bleeding<br>10. Eclampsia / convulsions<br>11. Hypertension (any type)<br>12. Breech or other abnormal presentation (Cormic / transverse)<br>13. Previous caesarean<br>14. Gestational Diabetes<br>15. HIV Infection<br>16. Fetal death<br>17. With no clinical or obstetric diagnosis<br>18. Another diagnosis (answer 89 then go to 91)<br>19. Medical complications (go to 90)
4	89	Which other diagnosis?	String format
4	90	Which medical complications?	String format
4	91	Was there a caesarean section indication on admission?	0. No 1. Yes (go to 130)
**Part 5**		**LABOUR CARE INFORMATION**	
5	92	Date of admission in labour ward	Date format
5	93	Time of admission in labour ward	Time format
5	94	**Labour:**	-
5	95	Medications/ methods used for labour induction	1. Oxytocin 2. Misoprostol 3. Other
5	96	Any companion present during labour?	0. No 1.Yes 9. Not registered
5	97	Prescription diet during labour:	0. Nil by mouth 1. Liquid diet 2. Another type of diet 9. Not registered
5	98	Prescription of bed rest during labour:	0. No 1. Yes
5	99	Prescription of intravenous lyquids during labour:	0. No 1. Yes (go to 101)
5	100	Placement of venous cannulation during labour:	0. No 1. Yes
5	101	Prescription of antibiotics during labour:	0. No 1. Yes
5	102	Shaving for birth (in hospital)?	0. No 1. Yes
5	103	Enema before delivery?	0. No 1. Yes
5	104	Role of professional that assisted labour	1. Medical doctor 2.Obstetric nurse 3. Nurse 4. Midwife
5	105	Was there record of partogram in the medical record?	0. No (go to 110) 1. Yes

**5**	**106**	Dilatation of the cervix was registered at the beginning of the partogram?	0. No (go to 108) 1. Yes
**5**	**107**	How many centimeters?	Number format
**5**	**108**	Was the number of times the cervix was checked for dilatation registered in the partogram?	0. No (go to 110) 1. Yes
**5**	**109**	How many times?	Number format
**5**	**110**	Prescription of synthetic oxytocin during labour?	0. No (go to 116) 1. Yes
**5**	**111**	**Prescription of oxytocin (Anotate the first prescription before delivery):**	-
**5**	**112**	Number of ampoules of 5UI/500 ml serum	Number format
**5**	**113**	No. of drops / min	Number format
**5**	**114**	Infusion rate ml / hour	Number format
**5**	**115**	Dilatation of the cervix in the administration of oxytocin (in centimeters)	Number format
**5**	**116**	Prescription of pain relief medication during labour (it is possible more than one answer)	1. No<br>2. Yes, opioids (MEPERGAN, meperidine, demerol or pethidine)<br>3. Yes, other (buscopam, dipyrone, hyoscine, etc.)
**5**	**117**	**Use of non-pharmacological methods of pain relief in labour:**	-
	**118**	Water in Shower	0. No 1. Yes
**5**	**119**	Bath tub	0. No 1. Yes
**5**	**120**	Massage	0. No 1. Yes
**5**	**121**	Birthing ball	0. No 1. Yes
**5**	**121.1**	Birthing stool	0. No 1. Yes
**5**	**122**	Rocking birth stool	0. No 1. Yes
**5**	**123**	Other	0. No (go to 125) 1. Yes
**5**	**124**	Specify other here	String format
**5**	**125**	Use of anesthesia/analgesia during labour:	0. No 1. Epidural 2. Spinal 3. Spinal + epidural (combined)<br>4. General
**5**	**126**	Rupture of membranes during labour/delivery:	0. No, it was broken before admission (go to 129)<br>1. Yes, spontaneous rupture<br>2. Yes, artificial rupture (made by professionals)<br>3. Yes, but unspecified how

**5**	**127**	Characteristic of amniotic liquid:	1. Clear liquid with no lumps<br>2. Clear liquid with lumps<br>3. Fluid with meconium<br>4. Bloody fluid<br>5. Purulent fluid / foul<br>6. Unspecified
**5**	**128**	Dilatation of the cervix at the time of rupture of membranes (in cm):	Number format
**5**	**129**	Is it registered in the medical record any of the following conditions?<br>**it is possible to have more than one answer**	1. Fetal distress during labour<br>2. Elimination of thick meconium<br>3. Fetal bradycardia (BCF <110)<br>4. Fetal tachycardia (BCF> 160)<br>5. Presence of DIP 2 (slowdown in cardiotocography)<br>6. No record of any of the above conditions
**Part 6**		**BIRTH CARE INFORMATION**	
**6**	**130**	Date of birth?	Date format
**6**	**131**	Time of birth?	Time format
**6**	**132**	If any companion was present during birth?	0. No 1. Yes 9. Not written in the medical record
**6**	**133**	Type of delivery?	1. Vaginal (including forceps)<br>2. Cesarean (go to 146)<br>(In case of twins with both vaginal and cesarean birth, complete with both types of birth) "
**6**	**134**	Use of forceps / vacuum extractor?	0. No 1. Forceps 2. Vacuum
**6**	**135**	The role of professional who assisted the birth?	1. Phisician<br>2. Obstetrician nurse<br>3. Nurse<br>4. Midwife<br>5. Nurse technician<br>6. Student<br>7. Other<br>9. Not written in the medical record
**6**	**136**	Position of women during birth:	1. Lying on your back with legs raised<br>2. Lying on one side<br>3. Sitting / reclining<br>4. in the bathtub<br>5. All fours support<br>6. squatting<br>7. standing up<br>9. Not written in the medical record

**6**	**137**	Time when the pregnant woman reached full dilatation (in partogram or medical records):	Time format
**6**	**138**	The duration of the second stage	Number format
**6**	**139**	Episiotomy	0. No 1. Yes
**6**	**140**	Occurrence of vaginal/perineal lacerations?	0. No<br>1. Yes, first degree<br>2. Yes, second degree<br>3. Yes, third degree<br>4. Yes, fourth degree<br>5. Yes, unkown degree
**6**	**141**	Ocurrance of vaginal/perineal suture or episiorrhaphy?	0. No 1. Yes
**6**	**142**	Kristeller manoeuvre?	0. No 1. Yes
**6**	**143**	Any complications during birth or immediate postpartum period:	0. No<br>1. Shoulder dystocia<br>2. Cord prolapse<br>3. Uterine rupture<br>4. Prolonged expulsion stage<br>5. Uterine atony<br>6. Retained placenta<br>7. Other (answer 144)
**6**	**144**	Which complication?	String format
**6**	**145**	Use of anaesthesia:	0. No<br>1. Epidural<br>2. Spinal<br>3. Spinal + epidural (combined)<br>4. General<br>5. Local<br>6. Pudendal nerve<br>9. Not written in the medical record
**Part 7**		**INDICATION FOR CAESAREAN SECTION**	
**7**	**146**	Obstetrician Information	

**7**	**147**	1st obstetrician Indication	01. Previous Cesarean Section<br>02. Cephalopelvic Disproportion (CPD)<br>03. Failure to Progress Through labour<br>04. Placenta previa<br>05. Placenta abrubtion<br>06. Fetal distress / Intrauterine Growth Restriction (IUGR)<br>07. HIV Infection<br>08. Breech presentation(sitting)<br>09. Cormic presentation (crossed)<br>10. Tubal ligation<br>11. Hypertension / Preeclampsia<br>12. Eclampsia<br>13. HELLP syndrome<br>14. Diabetes<br>15. Oligohydramnios<br>16. Twin pregnancy<br>17. Prematurity<br>18. Postmaturity (Prolonged Pregnancy)<br>19. Macrosomia<br>20. Failed induction<br>21. Malformation<br>22. Fetal death<br>23. Ruptured of membranes<br>[illegible]
**7**	**148**	Another. Which?	String format
**7**	**149**	2nd obstetrician Indication	Same as 147
**7**	**150**	Another. Which?	String format
**7**	**151**	3rd obstetrician Indication	Same as 147
**7**	**152**	Another. Which?	String format
**7**	**153**	4th obstetrician Indication	Same as 147
**7**	**154**	Another. Which?	String format
**7**	**155**	Anaesthesia type:	
**Part 8**		**MATERNAL NEAR MISS**	
**8**	**156**	**The woman had any of the following clinical alterations whilst in hospital:**	-
**8**	**157**	Acute cyanosis?	0. No 1. Yes
**8**	**158**	Agonizing breath (gasping)?	0. No 1. Yes
**8**	**159**	Respiratory rate (RR)> 40 or <6 ipm?	0. No 1. Yes
**8**	**160**	Shock?	0. No 1. Yes

**8**	**161**	Oliguria unresponsive to hydration and medications?	0. No 1. Yes
**8**	**162**	Coagulation disorder?	0. No 1. Yes
**8**	**163**	Jaundice in the presence of pre-eclampsia?	0. No 1. Yes
**8**	**164**	Seizures reentrant / complete paralysis?	0. No 1. Yes
**8**	**165**	Stroke?	0. No 1. Yes
**8**	**166**	Loss of consciousness longer than 12 hours?	0. No 1. Yes
**8**	**167**	Loss of consciousness associated with absence of pulse?	0. No 1. Yes
**8**	**168**	**The woman had any of the following labouratory abnormalities whilst in hospital:**	-
**8**	**169**	O2 saturation <90% for more than 60 minutes	0. No 1. Yes
**8**	**170**	PaO2/FiO2 <200 mmHg?	0. No 1. Yes
**8**	**171**	Creatinine> = 3.5 mg / dl?	0. No 1. Yes
**8**	**172**	Bilirubin> 6 mg / dl?	0. No 1. Yes
**8**	**173**	pH <7.1?	0. No 1. Yes
**8**	**174**	Lactate> 5?	0. No 1. Yes
**8**	**175**	Acute thrombocytopenia (platelets <50,000)?	0. No 1. Yes
**8**	**176**	Loss of consciousness associated with the presence of glucose and ketoacids in the urine?	0. No 1. Yes
**8**	**177**	**Did the woman receive any of the following treatments whilst in hospital:**	-
**8**	**178**	Continuous use of vasoactive drugs (dopamine, dobutamine, epinephrine)?	0. No 1. Yes
**8**	**179**	Hysterectomy after infection, sepsis or bleeding?	0. No 1. Yes
**8**	**180**	Transfusion> = 5 units of red blood cells?	0. No 1. Yes
**8**	**181**	Dialysis for acute renal failure?	0. No 1. Yes
**8**	**182**	Intubation and mechanical ventilation >= 60 minutes (not related to anesthesia)?	0. No 1. Yes
**8**	**183**	Cardiopulmonary resuscitation?	0. No 1. Yes
**Part 9**		**NEWBORN's INFORMATION - PART 1**	
**9**	**184**	Newborn's number in medical records	Number format
**9**	**185**	Live Birth certificate registry number	Number format
**9**	**186**	Gender:	1. Male 2. Female 3. Undefined
**9**	**187**	Birthweight (grams):	Number format
**9**	**188**	Gestational age by LMP	Number format
**9**	**189**	Weeks:	Number format

**9**	**190**	Days:	Number format
**9**	**191**	Gestational age by ultrasound scan	Number format
**9**	**192**	Weeks:	Number format
**9**	**193**	Days:	Number format
**9**	**194**	Gestational age by Capurro assessment	Number format
**9**	**195**	Weeks:	Number format
**9**	**196**	Days:	Number format
**9**	**197**	Gestational age by the New Ballard assessment	Number format
**9**	**198**	Weeks:	Number format
**9**	**199**	Days:	Number format
**9**	**200**	If caesarean birth, inform the indications written on the newborn' medical records	-
**9**	**201**	1st Indication for the C-section	01. Previous Cesarean Section<br>02. Cephalopelvic Disproportion (CPD)<br>03. Failure to Progress Through labour<br>04. Placenta previa<br>05. Placenta abrubtion<br>06. Fetal distress / Intrauterine Growth Restriction (IUGR)<br>07. HIV Infection<br>08. Breech presentation(sitting)<br>09. Cormic presentation (crossed)<br>10. Tubal ligation<br>11. Hypertension / Preeclampsia<br>12. Eclampsia<br>13. HELLP syndrome<br>14. Diabetes<br>15. Oligohydramnios<br>16. Twin pregnancy<br>17. Prematurity<br>18. Postmaturity (Prolonged Pregnancy)<br>19. Macrosomia<br>20. Failed induction<br>21. Malformation<br>22. Fetal death<br>23. Ruptured of membranes<br>24. Clinical complications<br>25. Not registered in the medical records<br>[illegible]
**9**	**202**	If another, specify here.	String format
**9**	**203**	2nd Indication for the C-section	Same as 201

**9**	**204**	If another, specify here.	String format
**9**	**205**	3rd Indication for the C-section	Same as 201
**9**	**206**	If another, specify here.	String format
**9**	**207**	4th Indication for the C-section	Same as 201
**9**	**208**	If another, specify here.	String format
**9**	**209**	Apgar score at 1 minute:	Number format
**9**	**210**	Apgar score at 5 minutes:	Number format
**Part 10**		**NEWBORN's INFORMATION - PART 2**	
**10**	**211**	Resuscitation in the delivery room	0. No 1. Yes
**10**	**212**	O2 inhaled	0. No 1. Yes
**10**	**213**	Mask ventilation with an Ambu bag	0. No 1. Yes
**10**	**214**	Orotracheal intubation	0. No 1. Yes
**10**	**215**	Cardiac massage	0. No 1. Yes
**10**	**216**	Drugs	0. No 1. Yes
**10**	**217**	Other	0. No 1. Yes
**10**	**218**	If another, specify here.	String format
**10**	**219**	Other procedures performed in the first hours after birth:	0. No 1. Yes
**10**	**220**	Upper airway aspiration?	0. No 1. Yes
	**221**	Gastric aspiration?	0. No 1. Yes
**10**	**222**	Vitamin K (Kanakion)?	0. No 1. Yes
**10**	**223**	Creed`s maneuver (manually extracting the placenta; drops of silver nitrate on newborn`s eyes and manual pessure of bladder)?	0. No 1. Yes
**10**	**224**	Hepatitis B vaccination?	0. No 1. Yes
**10**	**225**	The baby went to incubator?	0. No 1. Yes
**10**	**226**	The baby was hospitalized?	0. No, (go to 256) 1. Yes
**10**	**227**	Use of oxygen after birth:	-
**10**	**228**	O2 Hood	0. No 1. Yes
	**229**	Continuous positive airway pressure (CPAP)	0. No 1. Yes
**10**	**230**	Mechanical ventilation	0. No 1. Yes
**10**	**231**	Within 28 days of life was the baby on O2 therapy (any type)?	0. No 1. Yes 8. The baby was no longer in hospital

**10**	**232**	If the baby was born premature, when he completed 36 weeks of corrected gestational age was he still on O2 therapy (any type)?	1.Term newborn<br>2. No<br>3. Has not yet reached 36 weeks<br>4. Yes<br>5. The baby was no longer in hospital
**10**	**233**	Was there an indication for admission in neonatal intensive care unit:	0. No 1. Yes
**10**	**234**	Was the baby admited to NICU:	0. No 1. Yes
**10**	**235**	Use of surfactant:	0. No 1. Yes
**10**	**236**	Hypoglycemia (blood glucose less than 40) in the first 48 hours of life	0. No 1. Yes
**10**	**237**	Antibiotic use	1. No<br>2. Before 48 hours of life (early sepsis)<br>3. After 48hours of life (late sepsis)
**10**	**238**	Phototherapy in the first 72 hours of life:	0. No 1. Yes
**10**	**239**	Maximum level of bilirubin in the first 72 hours of life (mg / dl)	Number format
**10**	**240**	Congenital malformation?	0. No 1. Yes
**10**	**241**	Other diagnoses during hospitalization:	-
**10**	**242**	Transient tachypnea?	0. No 1. Yes
**10**	**243**	Hyaline membrane disease?	0. No 1. Yes
**10**	**244**	Meconium aspiration syndrome?	0. No 1. Yes
**10**	**245**	Pulmonary hypertension?	0. No 1. Yes
**10**	**246**	Seizure?	0. No 1. Yes
**10**	**247**	Necrotizing enterocolitis?	0. No 1. Yes
**10**	**248**	Toxoplasmosis?	0. No 1. Yes
**10**	**249**	Congenital rubella?	0. No 1. Yes
**10**	**250**	Herpes?	0. No 1. Yes
**10**	**251**	Cytomegalovirus?	0. No 1. Yes
**10**	**252**	Congenital syphilis?	0. No 1. Yes
**10**	**253**	Children exposed to HIV?	0. No 1. Yes
**10**	**254**	Other?	0. No 1. Yes
**10**	**255**	Specify others here	
**10**	**256**	Exclusive breastfeeding:	0. No 1. Yes

**10**	**257**	Other foods that the baby received during hospitalization	1. Water<br>2. Intravenous glucose/ Oral glucose<br>3. Expressed human milk<br>4. Infant formula<br>5. Parenteral nutrition
**10**	**258**	Outcome	0. The baby was still in hospital after 28 days<br>1. Discharged from hospital<br>2. Neonatal death<br>3. Transfered to another hospital (go to 260)
**10**	**259**	Date of discharge/ outcome	Date format
**10**	**260**	Hospital where the baby was transferred to (name - city - state):	String format
**10**	**260.1**	Reason for being transferred	String format
**10**	**260.2**	Date of transfer	Date format
**10**	**260.3**	Outcome from hospital where the baby was transferred to	0. The baby was still in hospital after 28 days<br>1. Discharged<br>2. Neonatal death
**10**	**260.4**	Date of hospital discharge/outcome from the hospital where he was transferred to	Date format
**10**	**261**	Death cause that was registered in the newborn medical record	1. Extreme prematurity (< 1000g)<br>2. Infection<br>3. Congenital Syphilis<br>4. Malformation<br>5. Respiratory complications<br>6. Others (answer 262)
**10**	**262**	If other, specify here	String format
**10**	**263**	Number of the death certificate registry	Number format
**10**	**264**	Baby's weight at hospital discharge, death or at 28 days old, if still hospitalized (in grams)	Number format
**10**	**265**	Comments:	String format
