# Supplementary material for: Birth in Brazil: national survey into labour and birth
Source: Reprod Health. 2012 Aug 22;9:15. doi: 10.1186/1742-4755-9-15 (PMC3500713; doi:10.1186/1742-4755-9-15)
Supplement: Additional file 3 — PHONE INTERVIEW FROM 43 TO 60 DAYS AFTER BIRTH full questionnaire. [file 1742-4755-9-15-S3.pdf]

PHONE INTERVIEW FROM 43 TO 60 DAYS AFTER BIRTH

Good evening, my name is_______ and I work for a research group called “Born in Brazil”,of the Oswaldo Cruz Foundation. I would like to talk to Mrs. __________.

Shortly after birth you participated in our research at the _________ maternity/ hospital. At that time you were informed that we would contact you by phone to know about your health and the health of your baby. Is it possible for you to answer some questions now? It will take no more than 10 minutes.

Part I - Identification of mother and baby and data from admission for birth

Name:

Age:

Type of pregnancy:

Type of Birth:

Name of Maternity/ hospital:

Date of birth:

Outcome of the mother:

Date of discharge of the Mother:

Name of the first Baby:

Outcome of the first baby at birth:

Outcome of the first Baby on the current date:

Date of discharge/ death of the baby:

ALWAYS READ ALL ALTERNATIVES and check the one ANSWERED BY INTERVIEWED. FOR SOME QUESTIONS IT IS POSSIBLE TO CHECK MORE THAN ONE ALTERNATIVE, IN THESE CASES IT IS INFORMED AFTER THE QUESTION.

<u>PART II - *Near Miss*</u>

The following questions are related to health problems that you could have had during the past pregnancy, birth or until 42 after birth.
(Don't ask which health problem she had. Just follow the questions)

**1.** Did you have any health problem during the past pregnancy, *birth or until 42 after birth?* 0. No ***(Go to part III)*** 1. Yes	|___|
**2.** Did you faint because of this health problem? 0. No 1. Yes	|___|
**3.** Were you admitted to hospital because of this problem? 0. No ***(go to question 9)*** 1. Yes 2. You were already in hospital	|___|
**4.** Did you stay in hospital for more than one week? 0. No 1. Yes	|___|
**5.** Did you have your uterus removed because of this health problem? 0. No 1. Yes	|___|
**6.** Were you transferred to a hospital with more resources during this hospitalization? 0. No 1. Yes	|___|
**7.** Were you admitted to ICU during this hospitalization? 0. No 1. Yes	|___|
**8.** Did you need machines to help you breath during this hospitalization? 0. No 1. Yes	|___|
**9.** Did you have high blood pressure during the past pregnancy? 0. No 1. Yes	|___|
**10.** Did you have seizures during the past pregnancy , birth or after birth? 0. No (***go to question 12)*** 1. Yes	|___|
**11.** Have you ever had seizures not related to pregnancy? 0. No 1. Yes	|___|
**12.** Have you had INTENSE vaginal bleeding (above normal) that soaked your clothes, bed or floor during the past pregnancy or after birth? 0. No ***(go to question 14)*** 1. Yes	|___|
**13.** Did you have a blood transfusion because of this bleeding? 0. No 1. Yes	|___|
**14.** Did you have fever <u>after birth</u>? 0. No ***(go to part III)*** 1. Yes	|___|
**15.** Together with this fever, did you have chills? 0. No 1. Yes	|___|
**16.** Together with this fever did you have bad-smelling vaginal discharge? 0. No 1. Yes	|___|

Part III. Satisfaction with care received in hospital

Now I am going to ask about your stay in hospital *__for birth__* *and about your satisfaction with it.*

**17.** When you were going to hospital for birth, how would you score the amount of time it took you to get there?<br>1.Very good (less time) 2. Good 3. Average 4. Poor 5. Very poor (more time)	|___|
**18.** In the admission for birth, how would you score the amount time since you arrived in hospital until you were assisted?<br>1.Very good (less time) 2. Good 3. Average 4. Poor 5. Very poor (more time)	|___|
**19.** In the hospital stay for birth, how would you score the respect the professionals had with talking to you and having you at the hospital?<br>1.Very good 2. Good 3. Average 4. Poor 5. Very poor	|___|
**20.** To receive a respectful treatment also means to have exams performed in a respectful way. In this hospital stay for birth, how do you score the respect the health professionals had with your intimacy during the physical exam and the overall assistance (I.E. during vaginal examination and birth assistance?)<br>1.Very good 2. Good 3. Average 4. Poor 5. Very poor	|___|
**21.** In the hospital stay for birth, how would you score the clarity in which the health professionals explained things to you?<br>1.Very good 2. Good 3. Average 4. Poor 5. Very poor	|___|
**22.** In the hospital stay for birth, how would you score the time provided to ask questions about your health and your treatment?<br>1.Very good 2. Good 3. Average 4. Poor 5. Very poor	|___|
**23.** In the hospital stay for birth, how would you score the possibility to discuss with the health professionals about the decisions that were made about the course of you labour and birth?<br>1.Very likely 2. Likely 3. Not likely or unlikely 4. Unlikely 5. Very unlikely	
**24.** In the hospital stay for birth, do you think you were a victim of maltreatment or any other kind of abuse/ violence by the health professionals, as: (***It is possible more than one answer)***<br>1.No<br>2.Verbal abuse (shouted with you or cursed you)<br>3.Psychological abuse (threatened you, humiliated you or refused to assist you or give you pain relief)<br>4.Phisical abuse (Push you, hurt you or undertook a painful vaginal examination)	|___| |___| |___|
**25.** In your opinion, the care you received for birth was:	|___|

1.Very good 2. Good 3. Average 4. Poor 5. Very poor	
**26.** In your opinion, the care and orientation you received after birth until discharge from hospital were:<br>1.Very good 2. Good 3. Average 4. Poor 5. Very poor	|___|
**27.** In your opinion, the care the baby received in the hospital he was born was: 1.Very good 2. Good 3. Average 4. Poor 5. Very poor	|___|

<u>Part IV– Maternal Morbidity and use of services</u>

Now I am going to ask about health problems that you might have had during the first two weeks after birth.

Attention: Only for mothers with vaginal birth (including forceps)<br><br>**28.** During the first two weeks after birth did you feel pain in the stitches you had in your perineum/ vagina?<br><br>0. No pain 1. A little pain 2. Strong pain 3. Very strong pain 4. Unbearable pain 8. No stitches	|___|
Attention: Only for mothers with Caesarean section<br><br>**29.** During the first two weeks after the C-section, did you feel pain in the surgery stitches?<br><br>0. No pain 1. A little pain 2. Strong pain 3. Very strong pain 4. Unbearable pain	|___|
**30.** During the first two weeks after birth did you feel pain in your breasts or nipples?<br><br>0. No pain 1. A little pain 2. Strong pain 3. Very strong pain 4. Unbearable pain	|___|
**31.** Lately, have you been feeling tired, with lack of energy?<br><br>0. No 1. A little tired 2. Tired 3. Very tired 4. Exhausted	|___|
Questions 32 to 35 are only for women discharged from hospital in the first week after birh. If not, go to part V.<br><br>**32.** Were you advised to seek for a postpartum check-up visit in a health service from 7 to 10 days after birth?<br>0. No 1. Yes	|___|
**33.** Did you seek any health service for this postpartum check-up visit?<br>0. No (***go to part V)*** 1. Yes	|___|
**34.** Did you have this postpartum check-up visit? 0. No (***go to part V)*** 1. Yes	|___|
**35.** When did you have postpartum check-up visit?<br><br>1. In the first 15 days after birth 2. After 15 days after birth 9. Don't remember	|___|

Part V- Newborn data and care

(In case of twins, apply this part for every newborn)

Now I am going to ask about the health of (name of the baby) from birth to today.

**36.** The (name of the baby) is living with you?<br>*1.* No, the baby died (***go to part VI)***<br>**2.** No, the baby is living with someone else **(ask the 37)**<br>***3.*** No, the baby is in hospital since birth ***(end the interview)***<br>***4.*** No, the baby the baby is in hospital ***(go to 38)***<br>5. Yes ***(go to 38)***	|___|
**37.** Could you answer more questions about (name of the baby)?<br>0.No ***(end the interview)*** 1. Yes	|___|
**38.** Do you know how much (name of the baby) weighted when he/ she was discharged from hospital of birth?<br>***(Doesn't know 9999)***	|___||___||___||___|g
**39.** When (name of the baby) was discharged from hospital of birth he/ she was receiving your breast milk only?<br>0. No 1. Yes ***(go to 41)***	|___|
1. **40.** Why not? ***(Do not read the alternatives)*** Public hospital in the same minicipality<br><br>1. Because She had a health problem<br>2. Because the baby had a health problem<br>3. Routine of the hospital (they gave him/her another milk)<br>4. She didn't have enough milk/ the baby didn't latch on<br>5. Because She didn't want to breastfeed<br>6. Other reason. Write here____	|___| |___| |___|
**41.** From yesterday morning to this morning, **(name of the baby)** was breastfed?<br>0. No 1. Yes	|___|
**42.** From yesterday morning to this morning, **(name of the baby)** had another type of milk?<br>0. No 1. Yes	|___|
**43.** From yesterday morning to this morning, **(name of the baby)** had water, tea or juice?<br>0. No 1. Yes	|___|
**44.** After **(name of the baby)** was discharged from hospital any *health professional* advised you to give her/ him any other milk apart from your breast milk?	|___|

0. No (***go to 46)*** 1. Yes	
**45.** Which *health professional* advised you to give **(name of the baby)** other milk apart from your breast milk?<br><br>1. Paediatrician 2. Other physician 3. Nurse 4. Community health agent<br><br>5. Other health professional – Write here_______________________________	|___| |___|
**46.** Have you seek for a health service to the first visit with a Paediatrician or nurse for **(name of the baby)** ? **(doesn't count urgent visits)**<br>0. No ***(go to 49)*** 1. Yes	|___|
**47.** Did **(name of the baby)** have this first visit?<br><br>0. No ***(go to 49)*** 1. Yes	|___|
**48.** When was this first visit?<br><br>1. In the 1st week of life 2. In the 2nd week of life<br><br>3. In the 3rd week of life 4. In the 4th week of life<br><br>5. More than one month old 9. Doesn't remember/ know	|___|
**49.** Has **(name of the baby)** received anti Tuberculosis (BCG) vaccination?<br><br>0. No 1. Yes 9. NSI	|___|
**50.** Has **(name of the baby)** received anti Hepatitis B vaccination?<br><br>0. No 1. Yes 9. NSI	|___|
**51.** Has **(name of the baby)** had the newbornscreening test?<br><br>0. No (***go to 54)*** **1.** Yes **9.** NSI (***go to 54***)	|___|
**52.** When was it performed?<br><br>1. In the 1st week of life 2. In the 2nd week of life<br><br>3. In the 3rd week of life 4. In the 4th week of life<br><br>5. More than one month old 9. Doesn't remember/ know	|___|
**53.** Have you had the results for the newbornscreening test?<br><br>0. No 1. In the 1st week of life 2. In the 2nd week of life<br><br>3. In the 3rd week of life 4. In the 4th week of life<br><br>5. More than one month old	|___|
**54.** Did **(name of the baby)** have any health problem after being discharged from hospital? ***(It is possible to check more than one alternative)***	

0. No<br>1. Turned yellow (jaundice)<br>2. Had an infection<br>3. Had a fever (not related to vaccination)<br>4. Lost too much weight<br>5. Had a reflux<br>6. Had a respiratory problem<br>7. Had diarrhoea<br>8. Other problem. Which? _______________________________	|___| |___| |___|
**55.** Did **(name of the baby)** need to be bathed in light after being discharged from hospital of birth?<br>0. No 1. Yes 2. NSI	|___|
**56.** After being discharged from hospital of birth, **(name of the baby)** was ever admitted to a hospital and stayed hospitalized for more than 24h as a result of a health problem?<br>00. No ***(End interview)*** Yes, How many times?	|___||___|
**57.** Which health problem? ***(It is possible to check more than one alternative)***<br>1. Turned yellow (jaundice)<br>2. Had an infection<br>3. Had a fever (not related to vaccination)<br>4. Lost too much weight<br>5. Had a reflux<br>6. Had a respiratory problem<br>7. Had diarrhoea<br>8. Other problem. Which? _______________________________	|___| |___| |___|
**58.** For how long did she/ he stay in hospital in the last admission?	**|___||___|days**<br>**|___| weeks**<br>**|___| months**

**59.** In which hospital did **(name of the baby)** stay in the last admission?<br><br>1. In the same hospital of birth<br>2. Public emergency hospital<br>3. Private emergency hospital<br>4. Public hospital in the same municipality<br>5. Public hospital in the another municipality<br>6. Private hospital<br>7. Other – write here: ____	|___|

If Q36 ≠ 1 (for all newborns– see twins questions); End interview

<u>Part VI – Data from baby`s death</u>

Only for babies who died after being discharged from hospital of birth (Q36=1)

**60.** When did the baby die?	____/____/____
**61.** What time?	|___||___|h<br>|___||___| min
**62.** Where was the baby when it happened?<br>0. Home 1. On the way to hospital<br>2. In hospital – name: ___________________________________	|___|
**63.** Did you receive the death certificate? 0. No 1. Yes	|___|
**64.** What was the main cause(s) of your baby`s death? ***It is possible to check more than one alternative)***<br>1. Prematurity<br>2. Respiratory problem/ disease<br>3. Heart problem/ disease<br>4. Infection<br>5. Congenital malformation<br>6. Diarrhoea<br>7. Problem in the blood<br>8. Other cause. write____	|___| |___| |___| |___|

This is the end of the interview. This research will help improve the quality of the assistance to the pregnant women, birth, post-partum period, as well as the assistance to the babies. Thank you very much!

Telephones: (21) 2598-2620 or (21) 2598-2621
